# A systematic review of neurophysiological sensing for the assessment of acute pain

**DOI:** 10.1038/s41746-023-00810-1

**Published:** 2023-04-26

**Authors:** Raul Fernandez Rojas, Nicholas Brown, Gordon Waddington, Roland Goecke

**Affiliations:** 1 Human-Centred Technology Research Centre, Faculty of Science and Technology, University of Canberra, Canberra, ACT Australia; 2 Faculty of Health, Queensland University of Technology, Brisbane, QLD Australia; 3 Australian Institute of Sport, Canberra, ACT Australia; 4 University of Canberra Research Institute for Sport and Exercise (UCRISE), University of Canberra, Canberra, ACT Australia

**Keywords:** Pain, Research data

## Abstract

Pain is a complex and personal experience that presents diverse measurement challenges. Different sensing technologies can be used as a surrogate measure of pain to overcome these challenges. The objective of this review is to summarise and synthesise the published literature to: (a) identify relevant non-invasive physiological sensing technologies that can be used for the assessment of human pain, (b) describe the analytical tools used in artificial intelligence (AI) to decode pain data collected from sensing technologies, and (c) describe the main implications in the application of these technologies. A literature search was conducted in July 2022 to query PubMed, Web of Sciences, and Scopus. Papers published between January 2013 and July 2022 are considered. Forty-eight studies are included in this literature review. Two main sensing technologies (neurological and physiological) are identified in the literature. The sensing technologies and their modality (unimodal or multimodal) are presented. The literature provided numerous examples of how different analytical tools in AI have been applied to decode pain. This review identifies different non-invasive sensing technologies, their analytical tools, and the implications for their use. There are significant opportunities to leverage multimodal sensing and deep learning to improve accuracy of pain monitoring systems. This review also identifies the need for analyses and datasets that explore the inclusion of neural and physiological information together. Finally, challenges and opportunities for designing better systems for pain assessment are also presented.

## Introduction

In 2020, the International Association for the Study of Pain (IASP) revised the definition of pain, which currently reads^1^: “An unpleasant sensory and emotional experience associated with, or resembling that associated with, actual or potential tissue damage.” This definition encapsulates that pain has sensory and affective elements, both nociceptive (physiological encoding and processing of noxious stimuli) and neuropathic (it can happen at any time without a pain-inducing event) pain experiences, and a cognitive element indicated in the anticipation of potential harm. In addition, the revised IASP definition notes that a “verbal description is only one of several behaviours to express pain.” Based on this definition and interpretation, for this review, one can argue that pain can be measured in multiple ways and in multiple contexts.

Pain can be thought as a construct that can be assessed using different approaches: self report, behaviour (e.g., vocalisations, facial expressions, body movement), and physiological activity^2^. Self reports, or patient reported measures, have been considered the gold standard in pain assessment in clinical practice. Self-reporting tools, such as the numerical rating scale (NRS) or the visual analogue scale (VAS), provide a fast and simple way to measure pain, require minimal effort to administer and are easily understood by the clinician and patient^3^. These metrics rely on the patients’ ability to assess and communicate their own pain experience. Another alternative is the verbal rating scale (VRS), which is sometimes used for individuals (e.g., young children, adolescents or adults with speech problems or learning disabilities) who have difficulty translating their pain experience into a numerical value, thus, this metric uses words to describe the magnitude of pain experience. A disadvantage of this metric is that patients may find the VRS difficult to answer, since the answers describing pain may be ambiguous and may not represent the best fit to their pain experience^4^. In addition, fluency in the language used for the VRS can be a barrier to effective assessment of pain^5^.

Behavioural measures can be used in individuals with impaired cognition or language skills, or in patients for whom self report is not possible or invalid. These tools capture facial expressions (including grimace, opened mouth, raising of eyebrows)^6^, vocalisations (e.g., crying, moaning, screaming)^7^, or bodily movements (e.g., posture, rigid or tense body, rest and sleep)^8^ as indicators of pain from the perspective of an external observer, e.g., nurses, doctors, or carers. Thus, these metrics are completely dependent on others to be attentive to nonverbal signs in pain, which represents a challenge since the trained observers must be able to reliably distinguish pain from a variety of other facial and bodily expressions^9^. A clear limitation of these type of metrics is observer error and bias, since there is a possibility that two trained observers might interpret behaviours differently. Other limitations of this measure include (1) individual differences in the expression of pain, which may be considerable from patient to patient; (2) the tendency of patients to alter their behaviour in the presence of an observer^10^; or (3) the inability of some populations to display signs of pain due to early developmental stage in infants or the use of sedatives or blocking agents that may mask pain behaviours^11^.

Physiological measures are an alternative when self reports are not available or as a complement to a clinical assessment. Physiological measures might be more accurate than behavioural measures for patients with intellectual disability or non-verbal patients^12^. Physiological measures of pain are based on the assumption that pain induces changes in autonomic activity of the nervous system and that these variations can be observed using different sensors that measure nervous system physiology. Studies of physiological responses in non-verbal patients, infants, and children in clinical settings often include methods of assessment observing derived cardiovascular and respiratory parameters, such as heart rate, blood pressure, respiration rate, oxygen saturation rate, skin sweating, and pupil size variations^13,14^. In this context, the use of sensors to measure physiological changes are of great importance, since their use can provide a precise, systematic, and simultaneous assessment of different physiological indicators.

The appropriate management of pain is an essential element of care. This ethical duty to treat pain was highlighted by the Declaration of Montreal, which calls for “access to pain management as a fundamental human right”^15^. To support this ethical duty, there is a need to obtain an objective, reliable, and accurate physiologic marker of pain that can assist clinicians to establish the most beneficial treatment for patients in pain. The field of pain management would benefit enormously from further advancement in objective, physiologic markers of pain^3^. To gain insight into how neurophysiological indicators can serve as valid measures of pain, it is important to understand the underlying mechanisms of how neurophysiological signals can be used to capture pain. To this end, we will discuss some of the most common aspects of the nervous system that provide insights into pain.

### Aetiology of pain

In the event of painful stimuli on the body, pain is mediated by processing in the nervous system. The nervous system consists of two subsystems, the peripheral nervous system and the central nervous system (CNS). The main function of the CNS is the integration and processing of sensory information in the body. The CNS consists of the brain and the spinal cord. Information regarding the effect of painful stimuli on the affected area (within the body or on extremities and trunk) is transported through the peripheral nervous system to the central and autonomic nervous systems by means of neural afferent pathways^16^. This process, in which the brain is informed of actual or potential tissue damage, is known as nociception. It is worth noting that in most cases, nociceptive stimulation (e.g., bruises, cuts, fractures) leads to pain; however, pain (e.g., phantom limb pain) can occur in the absence of any noxious stimulation. In this regard, nociceptive pain is often acute and brief in duration, and originates in response to a sufficiently intense stimulus.

Sensory pain receptors (also known as nociceptors) are sensory neurons that are attached to thin afferent nerve fibres (located at the skin, muscle, joints, bone, and viscera) and terminate in the dorsal horn of the spine^17^. Nociceptors help detect signals from potentially damaging stimuli in the body and can be activated when the stimulus is sufficiently intense. A nociceptor is a type of receptor with high threshold that respond to noxious thermal, chemical, or mechanical forces. This may be an intense mechanical stimuli such as cutting or pinching of the skin, intense heat or cold on the skin, or exposure to noxious chemicals^16^. When nociceptors are activated, the nociceptive signals are relayed to the spinal cord and transmitted to the thalamus within the CNS. The thalamus serves as central hub for sensory information before the signal is transported to different cortical areas of the brain responsible for integrating the information and response^18^. Contrary to well-defined sensory areas of the brain, such as the auditory cortex or visual cortex, there is no single pain cortex associated with pain perception^19^. Instead, there are multiple cortical and sub-cortical areas (also known as the pain matrix) that are associated with pain processing. These include the primary and secondary somatosensory cortex (S1, S2), anterior cingulate cortex (ACC), prefrontal cortex (PFC), insular cortex, nucleus accumbens, amygdala and thalamus^20,21^.

The peripheral nervous systems has two major subdivisions. The somatic nervous system is associated with predominantly voluntary activities, such as relaying instructions from the CNS to the muscles for voluntary movement. The autonomic nervous system is related to the regulation of involuntary, physiologic processes, such as regulating heart rate, blood pressure, or respiration^22^. The autonomic system is particularly associated with pain, since painful stimuli elicit an autonomic response (e.g., muscles will tighten, heart rate increases, skin temperature will fall) as an automatic defensive response in the body to avoid any further damage and to escape the painful stimulus. This autonomic response occurs irrespective of awareness or the pain experience^16,23^. In addition, the body will remember the noxious experience and can replicate the responses in the occurrence of similar events.

The autonomic nervous system (ANS) has two main branches that act the same time, the sympathetic (SNS) and parasympathetic nervous systems (PNS). These two regulate and control different autonomic functions in a number of vital organs. For instance, in case of painful stimuli, the SNS is involved in the body’s immediate response to danger (e.g., severe or acute pain); this autonomic reaction to pain is also known as the “fight or flight” response. The SNS activates a neurophysiological response that includes the regulation of blood flow, blood pressure, and vascular tone, and produces changes in blood pressure (BP), heart rate, sweat release, and pupil diameter^24^; thus, pain produces an increase of heart rate, blood pressure, oxygen intake, and sweat release, and leads to pupil dilation^25^. On the other hand, the PNS exhibits an inhibitory mechanism to pain that helps conserve and restore those neurophysiological responses exited during the sympathetic response. Among other functions, PNS slows the heart rate, decreases respiratory rate, and constricts pupils. These different functions make the measurement of pain complex.

Currently, there are sensing technologies that afford a non-invasive measure into the ANS and CNS. For instance, available metrics to measure changes in the ANS include, heart rate (HR) and heart rate variability (HRV) (both HR and HRV can be obtained either by electrocardiography (ECG) or photoplethysmography (PPG)), electrodermal activity (EDA), respiration (RESP), electromyography (EMG), and pupillometry. Similarly, there are non-invasive methods to measure activity in the CNS. Brain activity is measured by means of imaging techniques, such as electroencephalography (EEG), functional near-infrared spectroscopy (fNIRS), functional magnetic resonance imaging (fMRI), and magnetoencephalography (MEG). However, fMRI and MEG tend to be more intrusive^26^ and are, therefore, not considered in this review. The data obtained from each sensing technology must be subsequently analysed to enable an assessment of pain to be made.

The recording or measurement of physiological response require either real-time or post-collection processing and analysis. A range of processing and analysis techniques have been used in conjunction with the sensing technologies over many decades. In addition, there have been considerable advances in the use of tools, such as artificial intelligence (AI), to rapidly and more reliably determine an individual’s pain state. The development of AI tools combined with appropriate analytical models has made physiological sensors an ideal source of data to assist clinicians to make a more reliable and well-informed diagnosis of pain. With different advances in non-invasive sensing technologies and analysis approaches, which have emerged over the past decade, there is a need to explore the critical role they play in pain assessment.

Therefore, the purpose of this review is to integrate the literature on the different sensing technologies for the objective assessment of pain. This review also identifies how the information obtained by these sensors can be employed to develop autonomous mechanisms that can assist in estimating an individual’s pain. The contributions of this paper can be summarised as follows: (a) presenting a summary of the different sensing technologies that can be used in measuring an individual’s pain experience, which also includes a summary of the expected neurophysiological response (e.g., increase or decrease) during pain; (b) offering a detailed summary of analytical methods, including pre-processing, feature extraction and optimisation, and learning problem (e.g., classification or regression) results; (c) presenting a summary of the practical implications on the used of each sensing technology; and (d) a detailed discussion on the identified challenges and possible opportunities for designing better systems for pain assessment.

## Results

In this section, the study selection process including the steps considered for the selection of articles in this review is presented. In addition, the results for each of the research questions is presented in the following subsections.

### Study selection

**Fig. 1:**
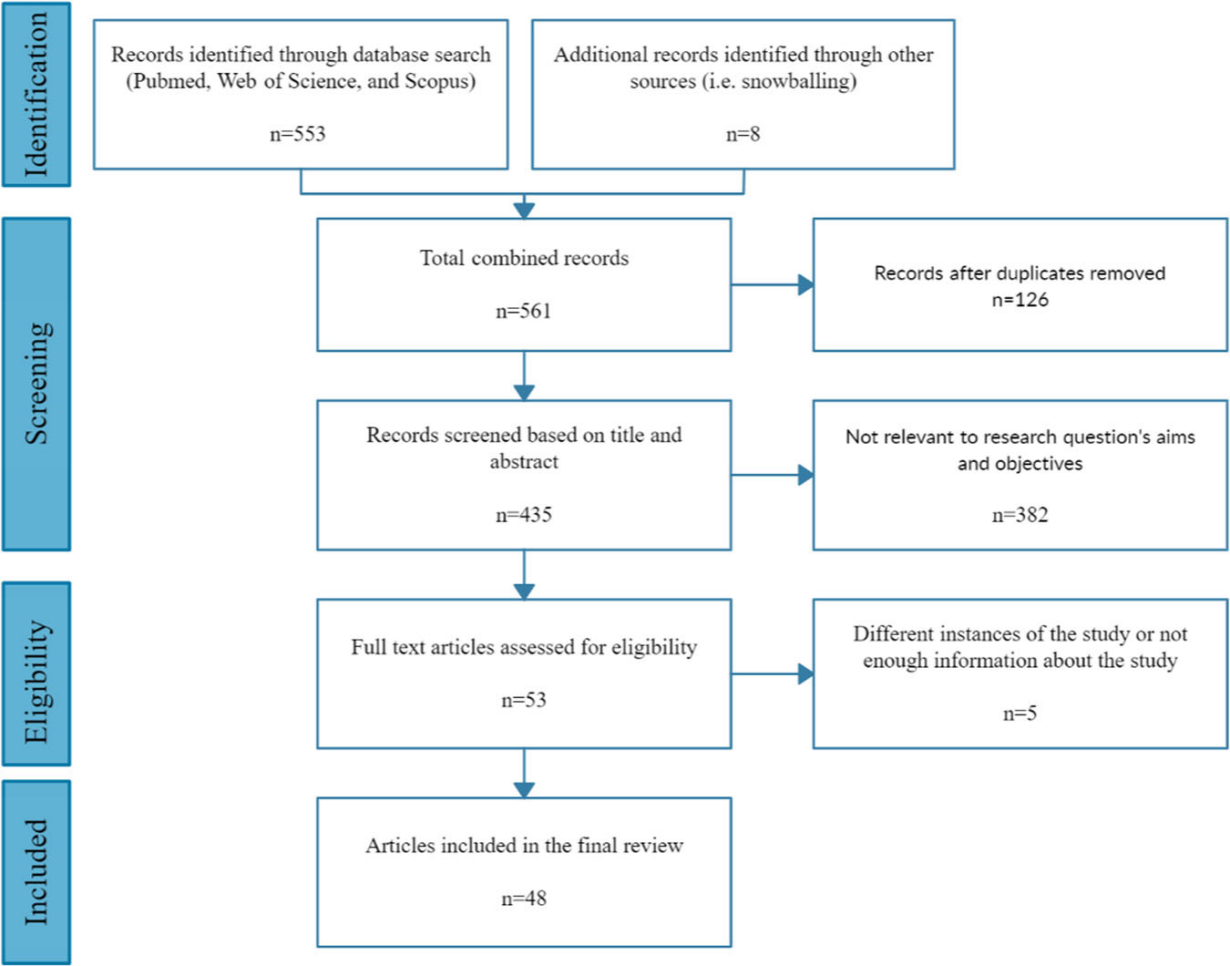
PRISMA flowchart. The diagram shows the study selection process conducted for the purpose of reviewing the use of sensors and machine/deep learning techniques for objectively assessing human pain.

Figure 1 presents the article identification and selection process. The search strategy retrieved 553 studies from the three databases and and additional 8 papers were manually included after searching in the reference lists of the identified studies. After removing duplicates found in different sources, 435 studies remained for further review. After screening the titles and abstracts against the inclusion criteria, 382 articles were discarded. The remaining 53 articles were read in their entirety. From those, a total of five papers were rejected for the following reasons: (1) they were different instances of the same study (*n* = 3), and (2) there was not enough information about the sensors and/or the analysis (*n* = 2). The remaining 48 papers were included in this review.

An overview of selected papers for this review is presented in Table 1. The summary presents information about the type of pain (e.g., electrical, thermal, postoperative, and sickle cell disease), the type of noxious stimulation used to elicit pain (e.g., laser, cold pressor test, thermode) and its anatomical location where the stimuli was applied (e.g., hand, arm, abdomen), and the population included (e.g., gender and age range) in each study. In addition, the sensor name to measure the neurophysiological response is also included. Based on the anatomical location, two categories can be observed in this summary: sensors that measure neural/brain activity (e.g., fNIRS, EEG) and sensors that measure other physiological activity (e.g., EDA, ECG, EMG, PPG)—it is worth mentioning that due to the exclusion criteria (non-invasive methods), surface EMG (sEMG) will be used hereinafter. Therefore, in the remaining of this paper, these two categories are referred as neurological and physiological sensors, respectively.

**Table 1:** Overview of reviewed studies in chronological order, their details and type of sensor used for pain detection.

Study	Type of pain	Stimuli	Location of stimulation	Population (F/M)	Age *μ* ± Std	Sensor	Year
^136^^◇^	Patient’s disease	–	–	11,428	65.8 ± −	ECG, RESP, SpO_2_, Pupil	2022
^160^^§^	Thermal & (heat) electrical	T: Thermode E: TENS	T: Left inner forearm E: Left index and middle fingers	127	18–50	ECG, EDA, sEMG	2022
^56^	Thermal (cold)	Cold pressor test	Right hand	32 (6/26)	21.25 ± 1.64	Pupil	2022
^31^	Electrical	TENS	Right forearm	16 (7/ 9)	25.6 ± 4.8	EDA	2021
^129^^♯^	Thermal (heat) & mechanical	T: Laser M: Pinprick	T: Dorsum of left hand M: Dorsum of right hand	T: 51 (25,26) M:2 (1,1)	T: 27 M: 58 ± 10.5	EEG	2021
^28^^†^	Thermal (heat)	Thermode	Right arm	87 (43,44)	18–65	EDA, ECG, sEMG	2021
^117^	Postoperative	Surgery	–	100 (56,44)	53.4 ± 12.5	PPG	2021
^85^	Thermal (heat & cold)	Thermode	Non-dominant hand	18 (3/15)	31.9 ± 5.5	fNIRS	2021
^98^	Physiotherapy	Pressure/tactile stimulation	Neck and arm	34 (23,11)	44 ± 15	EDA, sEMG, RESP, PPG	2021
^118^	Postoperative	TENS	–	25	–	PPG	2021
^46^	Electrical	TENS	Left & right arm	20 (9/11)	23–89 54.45 ± 17.44	ECG	2021
^42^	Electrical	TENS	–	20 (9/11)	23–89 54.45 ± 17.44	EDA	2021
^29^	Thermal (heat) & electrical	T: Thermal grill E: STMISOC stimulator	Right hand	25 T: 10 (4/6) E: 15 (9/6)	23–39 25 ± 4.8	EDA	2021
^83^	Thermal (heat)	Laser	Left hand	29 (9/20)	–	EEG	2020
^34^^⋆^	Thermal	–	Right arm	–	–	ECG, sEMG, EDA, RESP	2020
^63^	Thermal (cold)	Cold pressor test	Dominant hand	30 (13/17)	24 ± 3	EEG	2020
^64^	Thermal (heat)	Laser	Left & right hand	18 (8/10)	25 ± 3.5	EEG	2020
^32^	Thermal (heat)	Thermal grill	Right hand	10	23–39	EDA	2020
^52^	Postoperative	–	–	100	53.8 ± 12.4	PPG	2019
^69^	Thermal (heat & cold)	Thermode	Non-dominant hand	18 (3/15)	31.9 ± 5.5	fNIRS	2019
^43^	Sickle cell disease	–	–	20 (11/9)	20–66	PPG, EDA, SKT, GYRO, ACC, Steps	2019
^44^	Sickle cell disease	–	–-	29	–	PPG, EDA, SKT, GYRO, ACC, Steps	2019
^70^	Electrical	Neurometer	Left thumb	43 (0/43)	26.8 ± 5.6	fNIRS	2019
^35^^†^	Thermal (heat)	Thermode	Right arm	87 (44/43)	18–65	EDA, ECG, sEMG	2019
^96^	Thermal (cold)	Thermal probe	Left and right teeth	21 (13,8)	27.6 ± 3.5	fNIRS	2019
^41^	Thermal (heat) & electrical	T: Thermode E: TENS	T: Inner forearm on both arms E: fingertip of ring finger on both hands	30 (15/15)	F: 33 ± 11.9 M: 35 ± 8	ECG, RESP, EDA, sEMG	2019
^59^	Pressure	Algometer	Hand	9 (2/7)	–	EEG	2018
^36^^†^	Thermal (heat)	Thermode	Right arm	87 (43/44)	18–65	ECG, EDA	2018
^49^	Pressure	Periodontal probe		47 (13/34)	44–69	ECG, PPG	2018
^45^	Postoperative	Pressure/tactile examination	Abdomen	21 (5/16)	10–15	EDA	2018
^53^	Sickle cell disease	–	–	40	–	SpO_2_, BP, PPG, RESP, SKT	2018
^40^	Electrical	TENS	Tibialis anterior muscle of the right leg	6 (2/4)	22-25	BVP, ECG, EDA	2017
^66^	Thermal (heat & cold)	Thermode	Non-dominant hand	18 (3/15)	31 ± 5.5	fNIRS	2017
^84^	Thermal	Thermode	Left hand	25 (11/14)	24	EEG	2017
^161^	Thermal (cold)	Thermode	Left inner forearm	14 (4,10)	–	EEG, PPG, EDA	2017
^86^	Thermal (heat & cold)	Thermode	Non-dominant hand	18 (3/15)	31 ± 5.5	fNIRS	2017
^62^	Thermal (heat)	Thermode	Left forearm	30 (16/14)	20 ± 2	EEG	2017
^61^	Thermal (heat & cold)	Thermode & cold pressor test	Non-dominant hand	81 (45/36)	64.5 ± 12.5	EEG	2016
^37^^†^	Thermal (heat)	Thermode	Right arm	87 (43/44)	18–65	sEMG, EDA, ECG	2016
^72^	Thermal (heat)	Laser	–	96 (51/45)	21.6 ± 1.7	EEG	2016
^71^	Thermal(cold)	Cold pressor test	Hand	19 (10/9)	–	fNIRS	2016
^92^	Thermal (cold)	Cold pressor test	Non-dominant hand	13	–	EEG	2016
^38^^‡^	Thermal (heat)	Thermode	Right arm	87 (43/44)	18-65	sEMG, EDA, ECG	2015
^93^	Thermal (cold)	Cold pressor test	Dominant hand	17	23.22 ± 1.72	EEG	2015
^39^^‡^	Thermal (heat)	Thermode	Right arm	87 (43/44)	18-65	sEMG, EDA, ECG	2015
^33^	Pressure	Blood pressure cuff	Non-dominant arm	217 (120/97)	20 ± 1.80	ECG, EDA, SKT, PPG	2015
^94^	Thermal (heat)	Laser	Dorsum of right hand	23 (14/9)	19-35	EEG	2013
^103^	Thermal (heat)	Laser	Dorsum of left hand	29 (9/20)	17–25 22.2 ± 1.9	EEG	2013

Studies using datasets (◇ = MIMIC-III, † = BioVid Part A, ‡ = Biovid Part B, ⋆ = SenseEmotion, § = X-ITE, ♯ = Brain Mediators of Pain). Type of stimuli (T = thermal, M = mechanical, E = electrical).

*EDA* electrodermal activity, *fNIRS* functional near-infrared spectroscopy, *ECG* electrocardiogram, *Pupil* pupillometry, *EEG* electroencephalography, *sEMG* surface electromyography, *RESP* respiration, *PPG* photoplethysmography, *SKT* skin temperature, *GYRO* gyroscope, *ACC* accelerometer, *Steps* step counter, *SpO*_*2*_ oxygen saturation, *BP* blood pressure.

### Sensors used for pain assessment

**Fig. 2:**
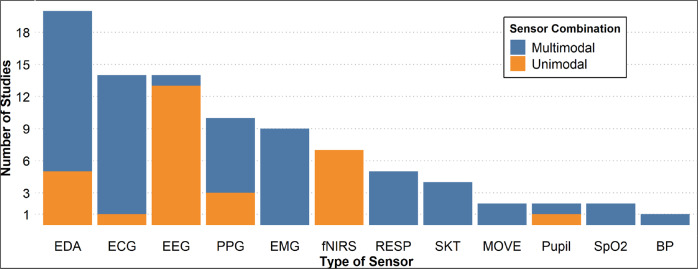
Summary of type of sensors used in the reviewed literature. EDA electrodermal activity, ECG electrocardiogram, EEG electroencephalography, PPG photoplethysmography, EMG electromyography, fNIRS functional near-infrared spectroscopy, RESP respiration, SKT skin temperature, MOVE gyroscope/accelerometer/steps, Pupil pupillometry, SpO_2_ oxygen saturation, BP blood pressure.

As described earlier, the two main categories of sensors used for assessing pain in the literature are: neurological and physiological sensors. Among the 48 studies, 27 studies (56%) assessed pain using physiological sensors, 20 studies (42%) assessed pain using neurological sensors, and only 1 study (2%) with a combination of neurological and physiological sensors. It is clear that the most popular sensors for the assessment of pain were EDA (*n* = 20), ECG (*n* = 14), and EEG (*n* = 14), while the least popular were movements sensors (accelerometer and gyroscope), Pupil, SpO_2_, and BP. Although, SpO_2_, BP, and pulse can be obtained from PPG, there was no indication of the type of sensor employed to obtain these metrics; therefore, we decided to maintain these metrics separately in the plot. It is also important to mention that, while the majority of studies used a single type of sensor (unimodal = 30), some of the retrieved studies used a combination (multimodal = 18) of two or more sensors for the assessment of pain. In addition, those studies using a multimodal approach, most of them employed physiological sensors (*n* = 17) only. Those studies using unimodal sensing, most of them (*n* = 20) used neurological sensors and only some (*n* = 10) used physiological sensors. Figure 2 presents the distribution of the reviewed studies along with their type of sensor modality (unimodal/multimodal).

In the following subsections, we report on the different sensors used of pain assessment. In addition, information regarding the effect of pain on the observed neurophysiological response (e.g., increase or decrease) with respect to each type of sensor and the location of the recording sensor on the participants of each study are also reported. This information is important for the interested reader who wants to explore the use of these sensors and replicate similar results.

#### Physiological measures

In the search for an accurate and objective method for pain recognition, various physiological signals have been explored as potential indicators for pain. These sensors are: Electrodermal activity (EDA), surface Electromyography (sEMG), Electrocardiogram (ECG), Respiration (RESP), Oxygen saturation (SpO_2_), Blood Pressure (BP), Electrocardiogram (ECG), Movement (MOVE), Skin temperature (SKT), Pupillary response (PUPIL), and Photoplethysmography (PPG). A summary of physiological sensors used in the assessment of pain is presented in Table 2.

**Table 2:** Summary of neurophysiological sensors used in the reviewed literature, including the anatomical location of recording sensor and the expected neurophysiological response to pain.

Sensor	Effect of pain (↑) Increase, (↓) Decrease	Location of recording sensor	Studies
EDA	(↑) Skin conductance (↓) Skin resistance	Palmar surface of index and ring fingers	^28,34–40^
		Palmar surface of index and middle fingers	^29,31–33,98,160^
		Palmar surface of two fingers	^41^
		Abode and below second knuckle of hand	^161^
		Wrist	^42–45^
sEMG	(↑) Trapezious muscle activity	Upper right trapezious	^28,34,35,37^
		Corrugator supercilii	^98^
	(↑) Corrugator supercilii muscle activity	Trapezious, corrugator supercilii, and zygomaticus	^38,39,160^
	(↑) Zygomaticus major muscle activity	Cheek and corrugator supercilii	^41^
RESP	(↑) Inspiration (↓) Breathing rate	Thorax	^34,41,98^
		–	^53,136^
SpO_2_	(↑) Oxygen saturation	–	^53,136^
BP	(↑) Systolic BP (↓) Diastolic BP	–	^53^
ECG	(↓) HRV (↑) HR	Right and left wrist	^46^
		Thorax	^28,34–41,49,160^
		–	^136^
		Wrist and ankle	^33^
MOVE	(↓) Movement (↓) Steps	Wrist	^43,44^
SKT	(↓) Skin temperature	Wrist	^43,44^
		–	^53^
		Fingertip of non-dominant hand	^33^
PUPIL	(↓) Pupillary diameter	Eyes	^56,136^
PPG	(↑) HR or pulse rate (↓) Blood volume pulse (↓) Pulse transit time	Left index finger	^40,49,52,117,161^
		Wrist	^43,44,118^
		–	^53^
		Left ring finger	^98^
		Thumb of non-dominant hand	^33^
EEG	N2 and P2 activation (↓) Alpha (↓) Beta (↑) Delta (↑) Theta (↑) Gamma	Full head	^61,62,72,83,84,94,103,129,161^
		Head circumference	^59,63,64,92,93^
fNIRS	(↓) HbR (↑) HbO	Somatosensory area	^66,69,85,86^
		Prefrontal and somatosensory area	^96^
		Prefrontal area	^70,71^

Among all of the physiological signals identified in the literature, electrodermal activity (EDA) was the most popular. In addition, EDA has been one of the most widely used physiological indicators in psychology, psychiatry, and psychophysiology research^27^. It is also refered as galvanic skin response (measuring changes in the skin’s electrical resistance) or skin conductance response (measuring changes in the skin’s electrical conductance)^28^. This type of sensor measures the changes in sweat gland activity which are affected by the sympathetic nervous system^29^. Increased sweating reduces (↓) skin resistance level (SRL), or in other words, the skin conductance level (SCL) increases (↑). When external stimuli are presented (e.g., visual, auditory, noxious, etc.), the skin momentarily becomes a better conductor of electricity^27^. These phenomena is normally recorded from palmar and plantar surfaces because of the higher density of glands in these areas^30^. In the searched literature, most studies used the palmar surface of two fingers (e.g., index and middle fingers) using electrodes^29,31–41^, while a small number of studies used a wristband to measure EDA on the wrist^42–45^.

Another popular physiological measure for indicating pain perception is electrocardiography (ECG). Since the autonomic nervous system regulates internal bodily functions involuntarily (e.g., those of the cardiovascular system), the heart plays a primary role. The heart provides a consistent flow of oxygenated blood (HbO), first by pumping deoxygenated blood (HbR) into the lungs (pulmonary circulation) for re-oxygenation, and then by sending HbO to the rest of the body (systemic circulation)^27^. The ECG signal is an indication of the heart’s contractile activity. The most significant parameters related to pain that have been derived from ECG signals are heart rate (HR) and heart rate variability (HRV)^46^. HR is a measure of the number (in heartbeats per minute or bpm) of contractions of the ventricles and HRV is a measure of the variance in time between heartbeats. An increase (↑) of HR and a decrease (↓) of HRV is associated with a stress response^47,48^. ECG can be measured on the thorax surface or in the limbs. Most surveyed studies used the thorax^34–41,49^, while other studies used either the wrists or the ankles^33,46^ to monitor the heart activity.

Electromyography (EMG) is another typical physiological recording method used to assess pain. This technique measures the electrical muscle activity originated by muscle contractions and propagated through the surface of the skin (i.e., surface EMG). In general, muscle contractions are associated with reflexes and actions characteristic of behaviour^27^. Electrical muscle activity is also a sign of general psychophysiological stimulation, as higher muscle tone is related to increased sympathetic nervous system activity and reduced somatomotor activity is primarily due to parasympathetic stimulation^39^. Although there is no a single muscle that can be targeted to be used as indicator of pain, in the area of affective computing, increased activity (↑) of the *trapezious muscle* is related to high stress^39^, while increase (↑) activity in the *zygomaticus and corrugator muscles* has been linked to an elicited response to unpleasant imagery^50^. In the reviewed literature, the trapezious muscle (located in the upper back, neck, near the shoulder area)^34,35,37–39,39^, and the zygomaticus major and corrugator supercilii muscles (located in the cheek and above brow, respectively)^41^ were used to measure muscle activity.

Another effective technique in detecting physiological changes is photoplethysmography (PPG). It is an optical method for detecting blood volume changes in the microvascular bed of tissue. It is frequently used to take measurements at the skin’s surface in a non-invasive manner, either by transmittance mode (e.g., a clip in the earlobe or finger) or by reflectance mode (e.g., a wrist watch)^51^. The PPG signal is multi-functional because it enables the extraction of many physiological indicators such as HR (or pulse rate), peripheral oxygen saturation (SpO_2_), and respiration rate reflecting the autonomic nervous system reaction^52^. The most common place to measure blood volume are the finger or the earlobe^27^. The thumb^33^ and the index finger^40,49,52^ of the non-dominant hand, and the wrist^43,44^ were the most popular anatomical locations in the reviewed literature. A decrease (↓) of blood volume pulse^33,40^, an increase (↑) in *HR*^43,44,49,52,53^, and an decrease (↓) of pulse transit time^33^ have been reported during different pain stimuli.

Skin temperature (SKT) is another metric for the identification of pain in the reviewed literature. The skin is a natural barrier that prevents the entry of external matter into the body and also allows the transmission of bodily fluids from the bloodstream to the exterior of the body^27^. The skin helps in the maintenance of water balance and core body temperature, which is achieved by generation of sweat (thermoregulation). Evidence suggest that generation of sweat changes the skin resistance, i.e., the higher the sweat, the lower the skin resistance^27^. Similar to EDA, this metric can be measured in palmar and plantar surfaces because of the higher density of glands in these areas^30^. In refs. ^43,44^, the skin temperature was measured in the wrist, while in ref. ^33^ it was measured in the finger tips. In addition, a decrease (↓) of local skin temperature has been observed after painful stimuli^53^.

Other various metrics such as respiration (RESP), oxygen saturation (SpO_2_), blood pressure (BP), movement (MOVE), and pupil changes (PUPIL) were also identified as possible indicators of pain. Respiration rate (also know as breathing rate) is a measure of the number of breaths per minute and it can be measured using an elastic belt worn around the thorax^34,41^. Respiratory changes often occur in response to pain, for instance, an inspiratory gasp with a subsequent breath-hold in response to acute pain^54^. In the reviewed studies, an increase (↑) in inspiration^34,41^ and a decrease of breathing rate were reported^53^. Although, RESP, SpO_2_ and BP can be obtained using PPG, in ref. ^53^ there was no indication of the method used to acquire these metrics; in this study, it was reported a decrease (↓) of breathing rate, an increase (↑) of oxygen saturation, and decrease (↓) of systolic and diastolic blood pressure. Movement metrics have been used to indicate changes in motor behaviour (e.g., slow movement, shorter duration, or fractionated movement)^55^. In ref. ^53^, a decrease (↓) in movement and number of steps as measure by 3-axial accelerometer and 3-axial gyroscope were reported. Finally, pupillary response to pain was investigated using cameras mounted on eye tracking glasses, it was reported that a decrease in pupillary diameter (i.e., dilation) was observed as a response to painful stimulation^56^.

#### Neurological measures

As human brain is the centre of any response to a certain stimulus, it is believed that neurological signals are highly correlated to complex integrative functions, such as sensory and motor integration^57^. In the reviewed literature, two types of neurological sensors were used to assess pain: (1) electroencephalography (EEG) and (2) functional near-infrared spectroscopy (fNIRS). Table 2 also presents a summary of the reviewed studies using neurological sensors.

In the reviewed studies, the most popular method of assessment of pain using neurological sensors was electroencephalography (EEG). EEG measures the brain’s electrical activity and pattern analysis of this activity is used to indicate neural activation associated with pain under certain frequencies. Spectral analysis is employed to decompose EEG signals into its constituting frequency components, between 1 and 60 Hz^58^. Typically, EEG data are partitioned into five bands (from slowest to fastest: delta, theta, alpha, beta, and gamma). The power spectral density (PSD) in each band is computed and used to compare the conditions being studied (i.e., pain vs. no pain). EEG is considered the most popular approach in the literature to objectively assess other cognitive states such as attention, cognitive workload, or vigilance.

EEG studies have identified correlations between pain and the power at different EEG frequency bands. Power in Delta band (1–4 Hz) has exhibited an increase (↑) after mechanical (pressure) pain^59^ and cold pressor test^60,61^. Power in Theta band (4–8 Hz) has also shown an increase (↑) after mechanical^59^ and thermal pain^62^. Power in Alpha band (8–12 Hz) was the most common indicator in the literature, the power in this band showed a decreased (↓) after cold pressor test^60,61,63^ and noxious laser stimulation^64^. Power in Beta band (12–30 Hz) showed an increase (↑) after heat stimulation^65^. Finally, power in Gamma band (30–45 Hz) exhibited an increased (↑) after heat stimuli^62,65^ and cold pressor test^63^.

The second approach to assess pain with neurological sensors is to measure activation of different brain regions using function near-infrared spectroscopy (fNIRS). This technique examines the levels of oxygenation (HbO) and deoxygenated (HbR) haemoglobin concentration in the cerebral cortex^66,67^. fNIRS is commonly used in this regard to measure the amount of HbO, due to its better signal-to-noise ratio than HbR, in a given brain region in response to a noxious stimulation^68^. Different studies have reported that increased (↑) levels of HbO in different cortical areas correlates with increased stimulation after heat and cold^66,69^, mechanical and electrical^70^, and cold pressor test^71^. Although, neither of these studies reported the use of HbR, it is well known that HbR has an opposite effect than HbO.

### Techniques for the analysis of sensor data in pain assessment

**Fig. 3:**
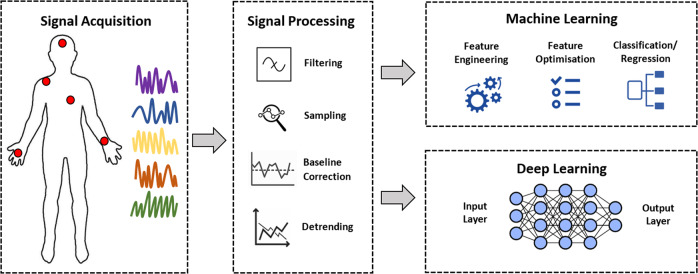
Data analysis pipeline of machine learning and deep learning models. After signal acquisition, the first stage in the data analysis pipeline is signal processing, followed by either the classical machine learning or deep learning pipelines.

Several studies have addressed the problem of automatic pain assessment using machine learning or deep learning methods by analysing both individual or multiple physiological signals. Traditional machine leaning methods rely on the design of manual feature extraction and feature optimisation to improve model performance. On the other hand, deep learning methods learn the intrinsic representations of the data to extract features directly from the data without the need of hand-crafted features. Despite of the method for feature derivation, two type of learning problems were identified in the reviewed literature: (1) classification, which qualitatively predicts the pain intensity by classifying trials into two (e.g., pain, no pain) or more levels (no pain, low, medium, high pain); and (2) regression, which quantitatively predicts the pain intensity as a continuous value (e.g., 0–10)^72^. In this section, we present the analytical methods used to decode pain from neurophysiological signals in the reviewed literature. Table 3 exhibits a summary of the analytical methods used in the reviewed studies. In addition, Fig. 3 presents the data analysis pipeline, this diagram is used to present the findings from the literature into four main categories: pre-processing, feature extraction, feature optimisation, and learning models (classification/regression).

**Table 3:** Summary of techniques used for the analysis of sensor data.

Sensors	Pre-processing	Feature extraction	Feature optimisation	Learning problem	Best results	Study
EDA	Downsampling (500–4 Hz) Moving median window (1 s) Low-pass filter (1 Hz)	Phasic component of EDA, spectral features time-varying index of sympathetic activity, modified time-varying index of sympathetic activity	–	Classification (N,H)(L,M,H) & Regression (0–10)	(N,H): G-SVM = 87% (L,M,H): G-SVM = 63% (1–10): M-RMSE = 0.78 M-MAE = 0.48	^31^
	Downsampling (128–4 Hz) Moving average window (1 s) Low-pass filter (50 Hz)	Statistical 14 features in total	GI	Classification (N,H)	RF = 86% with 4 features	^42^
	Downsampling (128–4 Hz) Moving median window (1 s) to remove artefacts Resampling to 2 Hz High-pass filter (0.01 Hz)	Phasic component of EDA, spectral features time-varying index of sympathetic activity, modified time-varying index of sympathetic activity	–	Classification (N,P)	RF = 84.3%	^29^
	Downsampling (128–4 Hz) Moving median (1 s) Resampling to 2 Hz	Spectral features time-varying index of sympathetic activity and modified time-varying index of sympathetic activity	–	Classification (N,P)	LR & LDA = 80% using TVSymp features P-SVM = 90% using MTVSymp features	^32^
	Low-pass filter (0.35 Hz) Downsampling (16–1 Hz)	Statistical	PCA	Classification (N,P)	L-SVM = 77.66%	^45^
ECG	Downsampling (500–250 Hz) Moving average window (0.45 s)	Temporal, statistical, and frequency 32 features in total based on HRV	GI	Classification (N,H)	G-SVM = 63.86% using 8 frequency-based features	^46^
PPG	Bandpass (0.5–10 Hz) Moving average window (30 samples) for signal smoothing	Spectrogram images	–	Classification (N,P)	CNN = 71.4%	^117^
	Empirical mode decomposition to derive respiration signals	Statistical features 10 features in total	GI	Classification (N,L)(N,M) (N,H)	(N,L): SVM = 81.41% (N,M): SVM = 80.36% (N,H): SVM = 79.48% in all cases with only 8 features	^118^
	Low-pass filter (8 Hz) High-pass filter (0.5 Hz) Moving average window to obtain vasoconstriction intervals (0.111 s) and heartbeat intervals (0.667 s)	Time and frequency 17 features in total based on HRV	2-class: Wilcoxon test & 4-class: ANOVA	Classification (N,P) & (N,L,M,H)	(N,P): DBN = 86.79% with 15 features (N,L,M,H): DBN = 65.57% with 17 features	^52^
Pupil	Pupil diameter velocity method to remove artefacts (blinks)	Temporal and statistical 11 features in total	GA	Classification (N,L,H)	ANN = 81% with 3 features	^56^
EEG	High-pass (0.1 Hz) Low-pass (250 Hz) Downsampling (1000–500 Hz) Notch filter (50 Hz) for line noise removal ICA for removing eye and muscle artefacts	Power *θ* − *α* (4–12 Hz), *β* − *γ* (12–45 Hz), high *γ* (60–100 Hz)	–	Classification (L, H)	L-SVM = 60%	^129^
	Band-pass filter (1–30 Hz) to remove high-frequency interference ICA to remove eye artefacts	Amplitude of the N2 wave, autoencoder-based, and PCA-based features	–	Classification (L,H)	LR = 74.6% using AE-based features	^83^
	Band-pass filter (1–100 Hz) to remove high-frequency interference	Power in *δ*, *θ*, *α*, *β*, *γ*	–	Classification (N,L,M,H)	ANN = 94.83%	^63^
	Band pass (1–64 Hz) to remove high-frequency interference Band-stop filter (49–51 Hz) to remove power line	Power in *δ*, *θ*, *α*, *β*, and multi-fractal detrended fluctuation analysis (MF-DFA)	–	Classification (N,P)	SBELM = 89.3%	^64^
	–	Power in *δ*, *θ*, *α*, *β*1, *β*2, *β*3, *β*4, and low *γ*	–	Classification (N,H)	L-SVM = 98%	^59^
	Band-pass filter (1–100 Hz) to remove high-frequency interference Notch filter (60 Hz) to remove power line noise ICA to reject eye artefacts Downsampling (1000–256 Hz)	Wavelet	–	Classification (N,P) & (Pain Score: 1–10)	(N,P): RF = 95.33% (1-10): RF = 89.45%	^84^
	High-pass filter (0.25 Hz) MARA Toolbox for automated artefact rejection	Power in *θ*, lower *β*, and *γ*	–	Classification (L,H)	G-SVM = 89.58% with theta, beta, and gamma bands only	^62^
	Band-pass filter (1–80 Hz) to remove high-frequency oscillations	Power in *δ*, *θ*, *α*, *β*	JMI	Classification (no responder, responder)	L-SVM = 65% with delta only	^61^
	Band-pass filter (0.01–100 Hz) to remove high-frequency oscillations ICA was used to remove eye artefacts	Intensity of the time-frequency spectrogram short-time Fourier transform	PLSR	Classification (L,H) & Regression (0–10)	L-SVM = 77% MAE (SVR) = 1.70	^72^
	Low-pass filter (0.5–30 Hz) to remove high frequency interference Downsampling from 200–128 Hz	Wavelet	–	Classification (N,P)	G-SVM = 82%	^92^
	Band-pass filter (1–85 Hz) to remove high-frequency interference	Wavelet	–	Classification (N,P)	KNN = 75.21% with delta band	^93^
	Band-pass filter (1–30 Hz) Baseline correction ICA to remove eye blinks and movements	Five coefficients from multiple linear regression	–	Classification (L,H) & Regression (0–10)	NB = 80.3% MAE (Linear Regression) = 1.821	^103^
	Downsampling (1000–512 Hz) ICA for correcting eye movements	Intensity of the time-frequency spectrogram using short-time Fourier transform	IG	Classification (L,H)	SVM = 83% with FCz or Cz	^94^
fNIRS	PCA to reduce extracerebral haemodynamics and systemic variables	Deep learning-based	–	Classification (L,H)	Bi-LSTM = 90.6%	^85^
	Band pass (0.01–0.3 Hz)	HbO and HbR signals		Classification (N,P) and (LP,RP,N)	(N,P): 3-layer CNN = 80.37% (LP,RP,N): 6-layer CNN = 74.23%	^96^
	Low-pass filter (0.16 Hz) to remove high-frequency oscillations PCA to reduce extracerebral haemodynamics and systemic variables	Time, frequency, wavelet 69 features in total	JMI	Classification heat (L,H) & cold (L,H)	G-SVM = 89.44% with 13 features from JMI	^69^
	GLM to reduce extracerebral signals Savitzky-Golay filtering to remove motion artefacts Low-pass filter (0.5 Hz) to remove high-frequency oscillations	Wavelet 80 features in total	–	Classification (N,P)	HB-LR = 81%	^70^
	Low-pass filter (0.16 Hz) to reduce extracerebral components	Wavelet using bag-of-words representation	–	Classification heat (L,H) & cold (L,H)	KNN = 92%	^66^
	Low-pass filter (0.16 Hz) to reduce high-frequency noise PCA to reduce extracerebral haemodynamics and systemic variables	Time, frequency, wavelet	–	Classification heat (L,H) & cold (L,H)	KNN = 88.33% with wavelet features only	^86^
	Low-pass filter (0.14 Hz) to remove high-frequency noise	Coefficients from functional data analysis (FDA)	RFE	Classification (L,H)	G-SVM= 94%	^71^
ECG, EDA	–	Tonic- and phasic-based features from EDA, statistical features from HRV 27 features in total	–	Regression (0–10) & Classification (N,H)	LR = 74.21% MAE (LSTM) = 1.05 RMSE (LSTM) = 1.29 in all cases with EDA only	^36^
ECG, PPG	ECG: linear detrend PPG: Svitsky-Golay filter for signal smoothing	ECG: frequency, wavelet features PPG: time, frequency, wavelet, Stress Index, ANSS Index 8 features in total	FFS	Classification (N,P)	RF = 70% wih 5 features	^49^
EDA, ECG, sEMG	sEMG: band-pass filter ECG: QRS-detection algorithm	Statistical 36 features (12 each)	–	Classification (7 intensities) Regression (7 intensities)	LSTM = 79.8% for phasic pain LSTM = 48.4% for heat tonic pain MSE (LSTM-SW) = 0.06 for phasic pain MSE (LSTM-SW) = 0.11 for tonic pain In all cases with EDA only	^160^
	sEMG: band-pass filter (0.1–250 Hz) ECG: band-pass filter (0.1–250 Hz)	Statistical 22 features in total	GI	Regression (0–10)	Subject-independent: MAE (G-SVR) = 0.93 RMSE (G-SVR) = 1.16 Subject-dependent: MAE (G-SVR) = 0.92 RMSE (G-SVR) = 1.13 In all cases with only 3 EDA features	^28^
	EDA: low-pass filter (0.2 Hz) sEMG: band-pass filter (20–250 Hz) ECG: band-pass (0.1–250 Hz) In all cases for removing artefacts	Deep learning-based features	–	Classification (N,H)	CNN = 84.57% using EDA only	^35^
	sEMG: band-pass filter (20-250Hz) ECG: linear detrend EDA: moving average window	Time, statistical, wavelet, HR- and HRV-based, and tonic- and phasic-based	–	Classification (N,H) & Regression (0–10)	RF = 85.7% MAE (RF) = 0.892	^37^
	sEMG: band-pass filter (20–250 Hz) ECG: band-pass (0.1–250 Hz) EDA: moving average	Time, statistical, frequency, and HR-based	FFS	Classification (N,H)	RF = 75%	^38^
	sEMG: band-pass filter (20–250 Hz) ECG: band-pass (0.1–250 Hz) In both cases to remove artefacts	Time, statistical, frequency 159 features in total	FFS	Classification (N,H)	G-SVM = 90.94%	^39^
EDA, ECG, PPG	PPG: band-pass filter (30–200 Hz) ECG: high-pass filter and moving average window EDA: moving average window and downsampling (256–128 Hz)	Statistical 36 features in total	GA, PCA	Classification (N,L,M,H)	G-SVM = 90.1%	^40^
	EEG: Band-pass (1–90 Hz) Noch Filters (60 and 120 Hz) ICA to remove movement and blinks PPG: Band-pass (0.05–50 Hz) Notch Filters (60 and 120 Hz) EDA: Band-pass (0.5 50 Hz) Notch Filters (60 and 120 Hz) Downsampling (500–25 Hz)	EEG: time-frequency bins (1 Hz) PPG: HR and HRV in frequency domain EDA: skin conductance response amplitude, skin conductance level, and skin conductance global amplitude	Statistical signficant EEG channels (*α* = 0.05)	Classification (N,P)	Sparse LR = 79%	^161^
ECG, RESP, SpO_2_, Pupil	Data are checked to fall within reference values, otherwise discarded Isolation outlier detection to remove outliers	Discrete values taken in random intervals (5–60 m)	–	Classification (N,H)(N,M) (N,L)	(N,H) AdaBoost = 63.71% with all sensors (N,M) Adaboost = 58.69% with HR, SpO_2_, and PUPIL (N,L) NN = 53.53% with HR, Pupil, RESP (N,H) for endocrinology Patients AdaBoost = 82.86% with all sensors	^136^
ECG, EDA, SKT, PPG	–	Time, statistical, frequency, HR- and HRV-based 27 features in total	–	Classification (L,H)	DFA = 84.7%	^33^
ECG, EDA, RESP, sEMG	sEMG: High-pass filter (20 Hz) Adaptive noise cancellation to remove power line and electrical pulse	Time and statistical	–	Classification (N,L,H)	ANN = 68.2%	^41^
	ECG: Band-pass (0.4–35 Hz) RESP: Band-pass (0.2–0.8 Hz) sEMG: Band-pass filter (0.05–25 Hz) EDA: Low-pass (0.2 Hz) In all cases for removing artefacts	Temporal, statistical, and frequency 311 features in total	–	Classification (N,H)	RF = 82.61%	^34^
PPG, EDA, RESP, sEMG, GRIP	sEMG: Downsampling (− to 256 Hz) BVP: Downsampling (– to 64 Hz) RESP: Downsampling (– to 8 Hz) EDA: Downsampling (– to 8 Hz) Moving average window to smooth the signal Signal decomposition to obtain phasic component GRIP: Downsampling (– to 75 Hz)	Time, frequency, wavelet 33 features in total	–	Classification (N,H)(L,H) (N,L)(L,M,H)	(N,H): AdaBoost = 94% (L,H): AdaBoost = 79% (N,L): AdaBoost = 85% (L,M,H): AdaBoost = 84%	^98^
PPG, RESP, SpO_2_, BP, SKT	–	Discrete samples from each sensor	–	Classification (Pain Score: 0–10)	L-SVM = 58.2%	^53^
PPG, EDA, SKT, GYRO, ACC, Steps	Moving average window	Statistical 64 features in total	FFS, BFE	Classification (N,L,M,H) & Regression (0–10)	SVM = 72.9% with 14 features from BFE RMSE (SVR) = 1.430 with 14 features from BFE	^43^
	Downsampling (1–0.1 Hz)	Statistical features 90 features in total	LASSO, Enet, RF, RFE	Regression (0–10)	RMSE (Stacked model) = 1.526 with 15 features	^44^

Feature optimisation (*GI* Gini index, *PCA* principal component analysis, *GA* genetic algorithm, *JMI* joint mutual information, *RFE* recursive feature elimination, *FFS* forward feature selection, *BFE* backward feature elimination). Learning Problem (*N* no pain, *P* pain, *L* low, *M* moderate, *H* high).

#### Pre-processing methods

The first step in the data analysis pipeline is pre-processing, which is generally done after signal acquisition. It is imperative to reduce or eliminate noise present in the captured data by implementing different pre-processing strategies, which are generally specific for each type of sensor. Noise (e.g., artefacts) can be defined as the unwanted changes that a signal may capture during data acquisition^73^. The signal within each sensor is typically a combination of the neurophysiological response to pain (or to the presented stimulus), environmental noise (e.g., power lines, electronic equipment), confounding factors (e.g., respiration or heartbeat in fNIRS data), or motion artefacts (e.g., body movement, poor sensor contact with the skin). When the noise components are stronger than the physiological data, the quality of the data may interfere with the analysis, its interpretation, and the validity of the data. The objective of pre-processing is to improve the quality of the signal by removing trends, filtering noise and artefacts, and in some cases, re-sampling the signal to reduce complexity in the analysis. Thus, pre-processing methods are an important step in the data analysis pipeline. In the following paragraphs, the processing steps for each individual sensor are presented.

EDA is vulnerable to several types of noise, including those generated from electronic noise, or movement between the skin and the recording electrodes. In the reviewed literature, the most widely used technique to remove signal artefacts and noise is using low-pass filters. This technique is able to smooth variations (e.g., movement, electrode pressure) in the signal^31,34,35,45^ and also to remove noise from the power source (50 Hz or 60 Hz)^42^ by attenuating unwanted (high) frequencies from the signal above a given cutoff frequency. A similar technique to reduce high-frequency components associated with artefacts is using moving windows, the moving media window^29,31,32^ and the moving average window^37,38,40,42,43^ are two examples of this technique. These windowing techniques eliminate extraneous data by computing the median and mean value within the window width (e.g., 5-s width), respectively. Another popular pre-processing technique used in the reviewed literature is downsampling the original signal. Downsampling is often used to reduce the data rate or the size of the data to speed up computation and reduce complexity^29,31,32,40,42,44,45^.

sEMG data are also susceptible to different types of noise. For instance, electromagnetic radiations from the power sources, high skin impedance, contamination due to relative motion of the sensor on the skin, cross-talk from nearby muscles, interference with the electrode cable or sensor detachment (also known as clipping), or ECG artefacts often observed from the muscles in the trunk^74^. Common motion artefacts, such as, electrode interface or electrode cable detachment, can be reduced by a better design of the system set-up^75^. On the other hand, inherent noise due to muscle cross-talk or instability of sEMG signals is believed to affect frequencies in the region of 0 to 20 Hz, which is the firing rate of the motor units^75^. In the reviewed literature, these kind of noise were attenuated with a band-pass filter to remove low and high frequencies from the signal^34,35,37–39,41^. The low-frequency cutoff (typically around 20 Hz) of the filter removes muscle cross-talk and inherent sEMG instability, while the high-frequency cutoff (typically greater than 200 Hz) removes high-frequency interference, such as movement artefacts, cable interference and clipping^76^.

ECG signals are mainly affected by sources of noise such as baseline drift, artefacts due to electrode motion, muscle contractions, and power source interference^77^. Baseline drift is a type of noise that presents an erratic up and down movement in the baseline signal, which is often a result of improper electrode placement or movement^78^. Muscle noise (i.e., EMG cross-talk) also affects the ECG signals, in particular in recordings involving human activity or exercise. In the reviewed studies, a moving average window^40,46^ or a linear detrend^37,49^ were used to smooth the signal and remove baseline drift (0.05–1 Hz). A band-pass filter^35,38,39^ was used to remove the baseline drift, movement (muscular and electrode) artefacts, and muscle cross-talk. A high-pass filter^40^ can be used to remove powerline interference (50 or 60 Hz), or motion artefacts and muscle cross-talk (ranging from 20 to 1000 Hz)^79^.

PPG is corrupted by various kinds of noise regardless of the type of the sensor design (reflectance or transmission). A typical PPG signal contains two main components, a large DC component due to the light absorbed when passing through the skin, tissue, and bone; and a small AC component due to the light passing through pulsating arteries caused by the heartbeat^80^. Common sources of noise in PPG signals are, powerline noise caused by electromagnetic interference (e.g., ambient light, computer screens), motion artefacts from sensor and/or body movements affecting the optical path, and physiological confounders (e.g., breathing). In the reviewed literature, a Savitzky-Golay filter^49^ was used for smoothing artefacts. A combination of low-pass and high-pass filter to remove high-frequency interference and to baseline drift (low-frequency), respectively^52^. Similarly, a band-pass filter^40^ was used to remove both low- and high-frequency noise. The application of a moving average window at two different periods were used to make the vasoconstriction and heartbeat intervals more salient in the PPG signal and remove motion artefacts^52^.

Other physiological signals such as, RESP, MOVE (gyroscope, accelerometer, and steps count), PUPIL, and SKT are also susceptible to noise. For instance, MOVE signals are highly affected by large voluntary and involuntary movement^81^. Similarly, respiration data are affected by movement artefacts. Blinks is the major source of noise in pupillometry. Skin temperature sensors are affected by movement artefacts or sensor detachment. In reviewed literature, a band-pass filter was used to compensate for movement artefacts in RESP data^34^, a moving average window was implemented to smooth the signal and reduce artefacts in the MOVE and SKT data^43^, and pupil diameter velocity method was used to remove high-frequency noise^56^.

EEG signals are also susceptible to various forms of noise, which affect their data quality. Often sweating and drifts in electrode impedance lead to slow changes in the measured potential, producing amplifier saturation, distortions, and lost data in the EEG signals. Similarly, muscle contractions typically affect the EEG signals above 100 Hz^82^. For these reasons, it is recommended to filter the frequencies below 0.01 Hz and above 100 Hz. In the reviewed literature, two types of filters were implemented to filter these type of noise. In ref. ^62^, a high-pass filter with a cutoff frequency of 0.25 Hz, while in refs. ^63,64,72,83,84^ a band-pass filter with a low- and high-cutoff frequency of 0.01–1 Hz and 30–100 Hz, were used respectively. Another common source of noise is the power line interference at 50 or 60 Hz, in ref. ^64^ a Butterworth band-stop filter (49–51 Hz) was used to remove the power source frequency interference. Another distinct source of noise is eye movements and blinks, in ref. ^63^ a visual inspection and a subsequent manual rejection was implemented; while, in refs. ^62,72,83,84^ independent component analysis (ICA) was used, and in ref. ^72^ the multiple artefact rejection algorithm (MARA) was implemented.

fNIRS data is generally contaminated by different sources of noise and pre-processing is required. Motion artefacts are generally seen as large spikes in the fNIRS data and in the reviewed literature, two hybrid methods were used to remove these artefacts: a discrete wavelet transform and statistical analysis^66,69^, and spline interpolation and Saviszky-Golay filter^70^. In addition, it is expected that high-frequency oscillations in the fNIRS data do not have a neural basis and are contaminated by cardiac pulsation (0.8–1.5 Hz) and respiration (0.16–0.6 Hz)^68^. Thus, in the work of refs. ^66,69–71,85,86^, a low-pass filter with a cutoff frequency below 0.16 Hz was applied to remove high frequency noise. Another distinct problem in fNIRS data is the inclusion of extracerebral hemodynamics from the scalp, skull, and systemic variables (e.g., blood pressure) that affect the data, in refs. ^69^^,^^66^ a de-noising procedure using principal component analysis (PCA) was implemented to identify and delete those components representing spurious signals in the fNIRS signals.

#### Feature extraction

The second step in the data analysis pipeline is feature extraction and its subsequent feature optimisation. A feature can be defined as an individual independent variable that serves as input data in a predictive/learning model. Feature extraction refers to the process to convert the sensor signals into numerical features (i.e., properties or characteristics) that can be used to create a predictive model using machine learning or deep learning. Features that better discriminate between input data from different classes (e.g., low, moderate, high pain), generally produce simpler and more accurate results. Since, features directly influence the performance of the predictive models and their results, thoughtful consideration to the feature extraction process (also called feature engineering) should be placed during the data analysis process. There are several feature extraction techniques that are applicable to most signals (e.g., statistical features), however, there are also some that are specific for each type of sensor (e.g., QRS-complex from ECG signals).

Statistical metrics are the most widely used features in many signal classification tasks. These kind of features often result in a good approximation to describe changes over time (e.g., summary statistics) across the values in the processing window (also called epoch)^87^. There are several popular statistical metrics including mean, standard deviation, range, kurtosis, or skewness. In the reviewed literature, in ref. ^39^ several statistical features were further divided into stationarity, entropy, linearity, variability, and similarity. In addition, statistical features were obtained from most sensors, PPG^33,40,43,44^, ECG^36–41,46^, sEMG^34,37–39,41^, EDA^33,34,37,38,38,40–44^, MOVE^43,44^, PUPIL^56^, RESP^34,41^, and SKT^33,43,44^.

Time-domain features are also popular features in time series analysis. These type of features are used to understand the shape of the signals within each window. Time-domain features are useful to find specific information about the signal, e.g., height (amplitude), length, or time to specific events (e.g., peaks, peak-to-peak amplitude). These metrics are important to compare the signals in different conditions (e.g., no pain and high pain) and also with respect to each sensor. Some of the most popular metrics obtained in the time domain include, maximum (max) and minimum (min) values, peak-to-peak values, time to peak, root mean square (rms), wavelength, slope, and area under the curve (AUC). Similar to the statistical features, time-domain features were obtained from most sensors in the reviewed literature, EDA^33,34,37–39,41^, PUPIL^56^, sEMG^34,37–39,41^, ECG^33,37–39,41,46^, RESP^34,41^, SKT^33^, EEG^83^, and fNIRS^69,86^.

Frequency-domain features allow to observe several characteristics of the signals that are not evident in the time domain. In signal processing, frequency refers to the number of cycles completed by a signal per unit of time, i.e., frequency is a measure of the occurrence of events in a specified time period^88^. When the information of interest repeats over time, frequency-domain analysis can be used to isolate oscillatory information within and across frequencies presented in the time series signals^89^. It is worth noting that, each type of sensor has distinctive characteristics in their frequency spectrum and therefore, appropriate analysis requires identification and processing for the unique frequency spectrum. For instance, EEG analysis is often based on the frequency decomposition of distinctive bands (e.g., *δ* (0.3–4 Hz), *θ* (4–8 Hz)), *α* (8–13 Hz), *β* (13–30 Hz), and *γ* (30–50 Hz)), which can be associated with specific functional characteristics. The most popular technique for frequency analysis is the Fourier Transform, which is mathematical technique that transforms a function of time (e.g., *x*(*t*)) into a function of frequency (e.g., *x*(*w*)), characterised by sine and cosines. Frequency-domain features include mean power in a specific frequency band, maximum power, or median frequency. In the reviewed literature, frequency-domain features were obtained from sEMG^34,38,39^, ECG^33,38,39,46,49^, EDA^33,34,38,39^, RESP^34^, PPG^33,49,52^, SKT^33^, EEG^59,61–64^, fNIRS^69,86^.

Time-frequency features are a useful technique in various forms of signal analysis. There are two main methods for time-frequency analysis, the short-time Fourier transform (STFT) and wavelets^88^. A clear property of time-frequency features is that they provide local information simultaneously, in both, time and frequency domain^90^. Time-frequency features allow to observe at which frequencies the signal oscillates and at which time these oscillations occur^91^, which can be helpful to study the frequency content during specific time periods or events (e.g., onset of pain). In the literature, wavelet-based features were computed from ECG^37,49^, PPG^49^, sEMG^37^, EDA^37^, fNIRS^36,66,69,86^, and EEG^84,92,93^, while STFT-based features were used from ECG^72,94^.

Other specialised features were also identified in the reviewed literature. Deep learning-based features, are obtained by deep neural networks able to automatically learn complex mappings (features) from input data (e.g., sensor data) to output classes or labels for classification problems or numeric values for regression problems, in an end-to-end manner^95^. In^83^ autoencoder-based features were obtained by training an autoencoder neural network, which compress the input EEG data into a lower-dimensional representation and then reconstruct the output from this representation. Similarly, in a fNIRS study^85^ and a multimodal (EDA, ECG, sEMG) study^35^, deep learning-based features were obtained from sensor data to automatically learn and extract relevant information in the pain data. Other specialised feature extraction techniques found in the literature are EDA-based features^29,31,32,36^, these features are based on the assumption that EDA signals have two salient attributes, the tonic (slow changing) and the phasic (fast changing) components; both tonic and phasic components are widely used to assess sympathetic arousal.

#### Feature optimisation

In the context of this review, feature optimisation refers to the process of reducing the size of the input variables (i.e., number of features) by means of feature selection or dimensionality reduction. In both cases, the aim of feature optimisation is not only to decrease the computational cost by reducing the number of features, but also to improve the performance of the predictive models by removing the irrelevant features or noisy data.

Feature selection is the process to reduce the number of features by identifying and selecting those features that have a strong relationship with the target variable (e.g., level of pain) and are believed to be the most informative for the design of predictive models. Feature selection methods can be organised into two main streams: model-based (wrapper and embedded methods) and model-independent (filter methods) methods. Wrapper methods search the space of possible feature subsets using the evaluation (e.g., training and testing) of a specific learning model; thus a search algorithm is “wrapped” around the model. Examples of wrapped methods in the literature are: recursive feature elimination (RFE)^44,71^, forward feature selection (FFS)^39,43,49^, back feature elimination (BFE)^43^, and genetic algorithm (GA)^40,56^. Embedded methods look for an optimal subset of features during the model’s construction, i.e., the learning models have their own built-in feature selection methods; embedded methods found in the literature are: least absolute shrinkage and selection operator (LASSO)^44^, random forest (RF)^44^, and Elastic Net (Enet)^44^. Filter methods, on the other hand, evaluate the features independently of any classification model by assessing the intrinsic properties of the data according to a certain criteria. Common objective criteria identified in the literature by filter methods are: Gini Index^42,46^, Information Gain^94^, Joint Mutual Information^61,69^, Wilcoxon Test^52^, Analysis of Variance (ANOVA)^52^, and Partial Least Squares Regression (PLSR)^72^.

Dimensionality reduction, on the other hand, refers to the methods that project features (input data) into a lower-dimensional feature space, resulting in entirely new input features^87^. Dimensionality reduction methods identified in the literature were based on principal component analysis (PCA)^40,45^, which works on the idea of finding a number of principal components that explain a specified amount of the variance in the data. In the machine learning literature, PCA can also be considered a feature extraction technique, since the identified principal components, that exhibit most of the variance, are considered a linear combination (a new set of features) of the original features. One of the limitations of dimensionality reduction is the fact that the obtained features are an abstract representation from the initial set of features, and this often affects the explainability of the learning models.

#### Learning models

Several studies have addressed the problem of automatic pain assessment using machine learning or deep learning methods. Traditional machine leaning methods rely on the design of manual feature extraction and feature optimisation to improve model performance. On the other hand, deep learning methods learn the intrinsic representations of the data to extract features directly from the data without the need of hand-crafted features. Despite of the method for feature derivation, two type of learning problems were identified in the reviewed literature, classification and regression. In this section, we summarise the type of learning problem based on the learning models as presented in Table 3.

Classification methods qualitatively predict the pain intensity by classifying trials into two (e.g., pain, no pain) or more levels (e.g., no pain, low, medium, high pain). This type of learning problem focus on discrete nominal outputs, however, a numerical variable (e.g., numerical value between 1 and 10) can be converted to an ordinal variable by dividing the range of the numerical variable into bins (e.g., 1–2, 4–6, 8–10) and assigning values to each bin, process commonly know as discretisation^87^. A popular machine learning model used in the literature is support vector machines (SVM)^43,94^, which often solves learning problems by using kernel functions to map the input data into higher-dimensional space in which the data can be separable (e.g., no pain or pain); kernel methods identified in the reviewed literature are Linear (L-SVM)^45,53,59,61,72^ and Gaussian (G-SVM)^31,39,40,46,62,69,71,92^, with G-SVM showing better results than L-SVM. Other popular classification methods in the literature are random forest (RF)^29,34,37,38,42,49,84^, logistic regression (LR)^32,36,70,83^, k-nearest neighbour (KNN)^66,86,93^, discriminant function analysis (DFA)^33^, sparse Bayesian extreme learning machine (SBELM)^64^, and artificial neural networks (ANN)^41,56,63^. In addition, deep learning models such as deep belief network (DBN)^52^, convolutional neural networks(CNN)^35,96^, and bi-directional long-short term memory networks (Bi-LSTM)^85^ were implemented to decode pain from sensor data.

Regression methods, on the other hand, quantitatively predict the pain intensity as a continuous value (e.g., 0–10)^72^. In the reviewed literature, regression models are used to obtain a continuous pain intensity based on the numerical values from the verbal numeric rating scale, where the subject or patient grades their pain sensation on a scale between 0 and 10^97^. Identified regression models using classical machine learning models are support vector regression (SVR)^43,72^, random forest (RF)^37^, and using deep learning models such as long-short term memory networks (LSTM)^36^. Another identified method is stacking or stacked model^44^, which is based on a combination of two or more regression models with the aim to harness the advantages of the individual models and obtain better performance than any single model in the assemble^87^.

### Practical implications

In order to understand the practical implications of each sensor, it is important to understand their advantages and disadvantages with respect to their use. In this section, we present a summary of the identified implications from the reviewed literature. The aim is not only to understand the main limitations, restrictions, and barriers but also, to identify the benefits and possible solutions to the application of these sensors in more realistic scenarios (e.g., clinical settings). Table 4 presents a summary of the limitations and advantages for each individual sensor.

**Table 4:** Summary of the practical implications from each sensor in their use for the assessment of acute pain as described in the reviewed literature.

Sensor	Limitations	Advantages
EDA	Physiological response can be caused by other factors than pain (e.g., surprise, stress)^31^	Well-established in the area of psychology, cognitive workload, stress, or affective computing^27^
	Stress is a strong confounding parameter, and a possible cause for false positives in pain detection^29^	High response to autonomic changes in the sympathetic nervous system^31,42,45^
	EDA cannot discriminate between stress and pain as both induce a positive response^32^	Small size, non-obstructive, and allows high mobility^29^
	It cannot differentiate source of pain (e.g., left or right side of body)^29^	It has shown better response than other physiological signals (e.g., ECG, sEMG) when used in a multimodal approach^35,36,98^
	Phasic EDA in response to a stimulus normally exhibits a latency of 1–3 s^45^	It can be used in an unimodal approach and has shown promising results in pain identification^29,31,32,42,45^
	Tonic signals can fluctuate within an individual, making it difficult to interpret^45^	Information about pain stimulation resides mostly in the phasic component of EDA^31^
	In the case of acute pain, the latency period may be confounded by anxiety and stress experienced in anticipation of the pain stimulus^45^	It can be used for quick and effective pain identification in clinical settings and in often difficult to assess populations (e.g., babies, children)^45^
sEMG	Facial expressions can be generated by other situations, so they can be misinterpreted^29^ or they can be feigned^28,29^	Increased muscle activity can be linked to increased activity in the sympathetic nervous system^39^
	Facial recording can be obstructive or interfere with other sensor technologies (e.g., pupillometry)^99^	Decreased somatomotor activity can be linked to parasympathetic stimulation^39^
	Increasing muscular tension in the trapezious muscle is associated with psychological stress^35,39^	Use of facial sEMG is more sensitive to muscle activation than camera-based monitoring^99^
	Facial expression-driven pain assessment can be very cumbersome in clinical settings^37^, in particular with oxygen masks^160^	Well-established in the area of sport science, rehabilitation, psychology, and affective computing^39^
	Facial sEMG have been found to be less significant than HR, BR, and GSR in the identification of acute pain^41^	Facial expressions of pain can serve as behavioural representation of pain^41^ and serve as direct mean to communicate pain^37^
	Facial sEMG is higly contaminated by eye movements and blinks^41^	
	Activity of zygomaticus muscle is linked to happiness^39^	
SKT	Environmental temperature and air movement is a confounding factor^33,162^	It can be easily integrated into a wireless wearable sensor with multiple sensors for continuous monitoring of pain^44^
	Changes in skin temperature can be generated by other situations (e.g., illness or fever)^100^	Skin temperature features have shown higher feature importance agains features from other vital signs^53^
	Stress is a strong confounding parameter in pain^33^	Available data can also be used to monitor well-being^43,100^
	Skin temperature is considered a relatively slow indicator of changes in pain^33^	Skin temperature serves as a surrogate marker of blood flow changes that result from vascular reactivity^33^
	It has not been used in isolation to measure pain^33,44^	
RESP	Chest strap sensors can be obstructive to other sensors attached to chest of the patient (e.g., ECG) and clinicians^41,52,101^	High response has been seen in cutaneous pain stimuli, even under anaesthesia^34^
	It has showed low effect against other metrics (e.g., temperature, BP, SpO_2_) to estimate pain^53^	It can be easily integrated into a wireless chest strap with multiple sensors to improve pain monitoring^41^
	Chest strap sensors can be uncomfortable for long periods of time and restrict movement, which makes it limited at measuring pain in clinical settings^52,101^	Available data can also be used to detect patient respiratory health, recovery, and monitor well-being^53^
	It has not been deployed in isolation^34,41,53^	
fNIRS	Respiration and cardiac pulse are confounding factors of fNIRS signals^69,86^	It allows measuring multiple cortical regions with large number of channels^66^
	Haemodynamic activity measured by fNIRS has a delayed response (2–5 s)^69,71,85^	fNIRS signals present higher spatial resolution than EEG, which allows better accuracy in identifying specific cortical areas^71,86^
	Large number of channels may not be suitable for practical applications in pain assessment^70,71^	fNIRS has the potential to differentiate the anatomical area where pain originates based on the cortical activity^66,96^
	Measuring occipital areas might be impractical for patients in supine position^70^	If patients are in a supine position, fNIRS can be used to measure cortical activity in the frontal area with the ability to avoid hair contamination, easier installation process, and more patient comfort^70^
		fNIRS has shorter set up time as compared to EEG, which makes it more clinic friendly for applications such as pain measurement or management in the clinic^71^
EEG	EEG data is highly affected by noise, e.g., eye movement, blinks, motion artefacts^64,94^	Well-established as a neuroimaging technique in the medical field for the diagnosis of epilepsy or sleep disorders^61,72^
	EEG systems need conductive EEG gel or saline solutions to increase the conductivity between the electrodes and the surface of the scalp^59^	If patients are in a supine position, it can be used to measure cortical activity in the frontal area with the ability to avoid hair contamination^70^
	EEG has lower spatial resolution than fNIRS^129^	EEG offers higher temporal resolution than fNIRS^59,83,84^
	Large number of electrodes are not suitable for practical applications as sensor preparation is time consuming^59^	It allows measuring multiple cortical regions with large number of channels^62,103^
	Measuring occipital areas might be impractical for patients in supine position^70^	EEG responses elicited by acute pain can serve to study the peripheral and central processing of nociceptive sensory input^103^
	EEG head caps are uncomfortable for long periods of time, which may not be suitable for practical applications in pain assessment^92^	A strong relationship between the N2 and P2 amplitudes in LEPs and the intensity of pain perception has been reported^103^
Pupil	Pupil size has showed lower classification accuracy than HR and SpO_2_ in the detection of pain^136^	There is not invasive contact between the patient’s skin and the data collection device^56^
	Data may be discontinuous or not available for long periods of time due to blinking^56^	Pupil size has exhibited better results than respiration rate in the detection of pain^136^
	Pain assessment based on pupillary response will be difficult to obtained in babies or unconscious patients^45^	Pupillary Diameter has been found more sensitive than heart rate or blood pressure during noxious stimulation^56,163^
ECG	HR can vary due to positive or negative emotions such as surprise, fear, or stress^33,34,40,49^	Well-established in the medical field to measure the rate and regularity of heartbeats^33,39,40^
	ECG signals have shown high intra- and inter-subject variability in pain responses, which may limit its usability^36,40,46^	ECG shows an strong response to sympathetic and parasympathetic activity^33,46^
	Variations in ECG signals in response to different pain levels are more difficult to differentiate in comparison to different pain levels versus baseline^46^	Time and frequency domain features based on HRV have been found specially useful during acute physiological changes (e.g., acute pain) for analysis of short time series (<1 min)^46^
	ECG signals have shown lower classification accuracy than other physiological signals^35–37,40^	ECG data can be used to monitor cardiovascular activity and overall well-being^40^
	It requires multiple electrodes, which makes ECG more obstructive and less convenient to be embedded into wearable devices^37,40,49^	
PPG	PPG signals are susceptible to motion artefacts (e.g., hand movement)^43^	Various parameters can be extracted, such as heart rate (HR) and heart rate variability (HRV), oxygen saturation (SpO_2_), blood pressure (BP), or respiration rate^40,52,104^
	PPG data can vary outside of pain when a patient is at rest due to factors including stress, excitement, and breathing^33,43^	PPG is less obstructive than ECG, as it can be placed anywhere on the body^40^
	HR-based features were found to be less important in pain assessment than EDA-based features^44^	It can be easily integrated into a wireless smart wristband with multiple sensors or a smart ring to improve pain monitoring^33,43,53,118^
	Pain quantification via HRV may not be useful in providing accurate assessment of the sympathetic nervous system^29^	Available data can also be used to detect cardiac conditions and monitor well-being^40,104^
	PPG data can be affected due to arousal or anxiety^117^	Decrease in BVP amplitude during pain compared to the baseline state implies peripheral vasoconstriction associated with arousal^33^
		BVP-based features have shown better classification accuracies than that of ECG in the detection of pain^40^
MOVE	Body movements can be originated due to daily activities and not due to pain^81^	It can be easily integrated into a smart wireless wristband with multiple sensors to improve pain monitoring^43,44^
	Body movement is negatively correlated with pain scores, which may reflect that patients in more pain typically move less frequently^44^	Available data can also be used to monitor well-being (e.g., physical activity)^43^
	However, lack of body movement can be originated due to other factors apart from pain such as, patient in rest, sleep, or anaesthetised^44^	For body movement measurements, acceleration and steps count have been identified as significant predictors for pain^44^

The use of EDA sensors is well-established in clinical practice and research. Applications using EDA include psycho-physiology, physical and cognitive stress, sleepiness, or affective computing^33^. EDA signals are modulated by autonomic changes in the sympathetic nervous system^31,42,45^, which not only drives elements of pain, bu also drives elements of human behaviour, cognitive states, or emotion^31,33^. For instance, there is a well-known association between EDA and emotional arousal, as the electrodermal activity changes in response to the emotional state (e.g., stressed, happy, sad). However, this high sensitivity to sympathetic function makes the discrimination between pain and emotional states difficult to accomplish^31^; this is a possible cause of false positives in pain detection^29^. EDA has exhibited promising results when used in isolation^29,31,32,42,45^ and has showed better results when compared with other sensors, such as, sEMG and ECG^35,36^, and RESP, BVP, and EGM^98^.

sEMG sensors are used to measure the electrical activity of muscle contractions propagated through the skin. Two main anatomical regions were used, the trapezius muscle (upper back of the torso)^35,37,38^, and the facial muscles (corrugator supercilii, zygomaticus major, risorious, orbicularis oculi, levator labii superiors)^39,41^. A clear disadvantage in the use of sEMG sensors to measure pain is that changes in muscle tone are highly associated with different affective states (e.g., stress, happiness, or anxiety)^35,39^. An advantage to use facial sEMG is that facial expressions serve as a direct means to communicate pain to other people, since individual’s affective state can be obtained by observing the face^37^. Pain assessment by means of facial expressions implies continuous tracking of a patient’s face, which can be difficult and cumbersome in clinical settings using cameras^37^. A possible solution is to use facial sEMG as it is more sensitive to muscle activation than camera-based monitoring^99^.

The use of SKT sensors to measure the temperature of the human body has been widely used in clinical assessment. In pathological conditions (e.g., locomotor, vascular, or malignant diseases), skin temperature serves as a valuable diagnostic information and well-being^43,100^. A clear advantage of SKT sensors to measure pain is that, SKT can be easily integrated into wireless wearable sensors with other sensors, which allows continuous monitoring^53^. Features from SKT data have shown higher feature importance against features from other vital signs (BP, SpO_2_, Pulse, and RESP)^53^. The main disadvantage of SKT sensors is that skin temperature is markedly affected by environmental temperature an air movement^33,100^. In addition, skin temperature has shown a rapid change due to emotional stress, which can confound data interpretation^33^.

RESP sensors are commonly used in clinical settings to monitor a patient’s respiration for intervention or diagnosis. In the reviewed literature, respiration data were obtained using chest straps^34,41^. An advantage in the use of RESP sensors to monitor pain is that multiple sensors can be integrated with the chest strap to monitor simultaneously different physiological signals (e.g., ECG, SKT)^41^. Data obtained from continuous respiration can also provide evidence on a patient’s respiratory health and recovery^53^. In addition, increased respiratory response has been observed in cutaneous pain stimuli, even under anaesthesia, which allows pain recognition while patients are under surgery^34^. On the other hand, a clear disadvantage in the use of chest strap is that they are prone to slippage (leading to inaccuracies), can be cumbersome to wear for long period of times (leading to uncomfortable patients), and can be also obstructive to other sensors and to clinicians^41,101^. Finally, this type of sensor has not been used in an unimodal approach, and it has showed low effect against other metrics (e.g., temperature, BP, SpO_2_) to estimate pain, while used in a multimodal approach^53^.

fNIRS systems provide a method for non-invasive monitoring of brain dynamics. These systems are used in different clinical settings as a neuroimaging technique in the field of neuroscience. The use of several sensors affords monitoring of different cortical areas simultaneously. fNIRS are safe to conduct brain monitoring in prolong time intervals^66^. fNIRS offers superior spatial resolution than EEG, which allows better accuracy in identifying specific cortical areas responding to changes in pain^71,86,102^. Its superior spatial resolution has the potential to identify the anatomical area where pain originates, based on the cortical activity^66^. However, the use of large number of channels is not practical for clinical applications, since some cortical areas (e.g., occipital, temporal, or parietal) are not accessible for continuous monitoring while the patient is in a supine position^71^. In these cases, a possible solution is to focus on the frontal area, with the additional advantage to have less hair contamination^70^. Other factors to consider when using fNIRS systems is that, fNIRS data are affected by cardiac pulse and respiration signals^69,86^, and the haemodynamic activity measured by fNIRS presents a temporal delay from the onset of the neural activity^69,71,85^.

EEG is also considered a brain imaging technique that allows non-invasive monitoring of neural activity. EEG systems measure the electrical activity of the brain. It has been widely used in the medical field in the diagnosis of epilepsy or sleep disorders^61,72^. EEG has higher temporal resolution than fNIRS, which affords a faster monitoring response (millisecond-scale)^59,83,84^. Using large number of EEG electrodes (e.g., 64, 128 electrodes) permits measuring the entire scalp^62,103^. A clear disadvantage of these systems is the need of conductive EEG gel or saline solutions to increase the conductivity between the electrodes and the surface of the scalp, which is generally time consuming^59^. Wearing an EEG head cap for long periods of time tends to be cumbersome and uncomfortable^92^. In addition, EEG is highly affected by blinks and eye movements, which requires different cleaning procedures^64,94^.

Pupillary response has been used extensively in human-computer interaction, attention monitoring, driver drowsiness, and cognitive workload^56^. Measures of pupil response can be obtained with cameras embedded in eyeglasses (e.g., Tobii Glasses). Thus, there is not invasive contact between the sensors and the skin, which has the potential to be less uncomfortable for the patients^56^. In addition, eyeglasses allow high mobility and non-obstructive application, as compared to external cameras^56^. However, an evident disadvantage is that patients must have their eyes open, which limits their use in unconscious patients or while sleeping. In addition, data cannot be available for long periods of time, or cannot be completely continuous due to blinking.

ECG sensors allow to measure the electrical activity of the heart on the skin surface. ECG has been widely used in the medical field to measure the rate (e.g., HR) and regularity of heartbeats (e.g., HRV), as well as the presence of any damage to the heart, and the effects of drugs or devices used to regulate the heart, such as a pacemaker^33,39,40^. In addition, ECG data can be used to monitor cardiovascular activity and overall well-being^40^. ECG shows an strong response to sympathetic and parasympathetic activity^33,46^. However, ECG physiological response can vary due to positive o negative emotions such as surprise, fear, or stress^33,34,40,49^. Another limitation is that ECG signals have shown high intra- and inter-subject variability in pain responses, which may limit its usability^36,40,46^. Also, ECG signals have shown lower classification accuracy than BVP and EDA signals^40^, and than EDA and sEMG signals^35–37^. Due to the number of leads used for ECG electrodes, ECG measures tend to be more obstructive and less convenient to be embedded into wearable devices^37,40,49^.

PPG is an optical method to measure variations of blood circulation. PPG offers multiple physiological indicators from both cardiac variations in blood volume (e.g., BVP) that arise from heartbeats, and from respiration and thermoregulation^104^. PPG signals offer an insight into the activity of the sympathetic nervous system^40^. PPG sensors can be easily integrated into a smart wristband with other physiological sensors^33,43,53^. PPG sensors can be placed anywhere on the body, with the finger as the most common location in the reviewed literature^40,104^. However, PPG signals obtained from a finger clip are susceptible to motion artefacts, e.g., hand movements, in and out of the bed or chair, or use of restroom^43^. Although, BVP-based features have shown better classification accuracies than that of ECG^40^, HR-based features were found to be less important in pain assessment than EDA-based features^44^. A clear confounding factor is that PPG data can vary outside of pain when a patient is at rest based on factors including stress, excitement, and breathing^33,43^.

Body movement (MOVE) can also serve as an important indicator in automatic pain estimation using sensors. An advantage of the movement sensors (e.g., accelerometers, gyroscopes) is that they can be easily integrated with other physiological sensors into wearable sensors for continuous monitoring^43,44^. In addition, body movement data can be also used to monitor well-being and identify early sings of health conditions related to sedentarism and lack of exercise in patients. Body movement measures such as number of steps and accelerometer information have shown a negative correlation with pain scores; this might reflect the fact that patients in more pain move less frequently^44^. However, lack of body movement can be originated due to other factors such as sedation, rest, or sleep^44^.

## Discussion

After a thorough survey of the literature, the challenges and future opportunities in the use of neurophysiological sensors for the assessment of acute pain are discussed in this section. Significant research is currently being done in the fields of sensor design, signal processing, time series analytics, and machine learning and deep learning. However, these efforts alone are not enough to solve a complex problem such as pain assessment. Thus, we should consider the challenges and limitations of the current state of the art methods and identify possible opportunities, which can make possible a smooth integration of the available methods and techniques for practical real-time applications. In this context, we highlight several challenges in the assessment of acute pain, and propose opportunities that can help us move closer towards the development of a bedside real-time monitor of pain. With that in mind, we present, in the following subsections, the challenges and opportunities with respect to each research question.

### Sensors to measure physiological changes in pain

Assessing pain based on a single sensor modality has major limitations. Sensor *reliability* is an important factor to consider when using a single sensor, as sensor failure reduces not only the quality of data, but also it causes loss of the physiological signal being measured^105^. In the medical field, data reliability is imperative to assess the clinical situation of the patient, and failing to provide reliable data might affect patient care and lead to patient deterioration^106^. For instance, the deployment of a pain monitoring system based on pupillometry alone will impact the reliability of the system, since the patient will be left unchecked while sleeping. Sensor *uncertainty* is another element to consider, interference (e.g., electrical noise) or confounding variables can negatively influence the physiological data and make it more susceptible to errors. For example, in the use of fNIRS sensors to measure the haemodynamic response, the fNIRS signals are often contaminated by superficial tissue (scalp and skull) and by other physiological indicators such as respiration or heartbeat^68,107^ that interfere with the expected signal from the cerebral cortex. In these cases, the use of a single sensor modality will produce noisy data, since all fNIRS sensors will be affected by the same source of noise. In addition, sensor *sensitivity* to a specific autonomic function (e.g., respiration, heart rate, pupil size) is another factor to consider. The use of a single sensor modality to monitor a single physiological parameter will limit the understanding of pain. Pain evokes multiple simultaneous neurophysiological signals that can offer a better comprehension of pain^39,85^. Although, using multiple sensor modalities will help solve some of the limitations of single sensor modality, the use of multiple sensor modalities must be balance against the ease of setup and application in a clinical setting.

The use of multimodal sensors for the assessment of pain presents several opportunities. Sensor *complementarity* is an important property of multimodal systems in which each sensor modality contributes to the whole system with specific information that cannot be obtained from any other single modality in the setup^108^. In the event of pain, multiple neurophysiological signals are triggered and by using different sensors modalities, it is possible to provide a different dimension of pain that will allow the system to obtain a more complete assessment of pain. For instance, fNIRS and EEG can complement each other to obtain a better assessment since EEG has higher temporal resolution but has lower spacial specificity, while fNIRS presents better spatial resolution yet lacks time precision due to its delayed haemodynamic response. Another advantage of multimodal systems is improved *observability*. Including multiple sensor modalities improves the diversity of data to monitor the physical measure. For example, combining measures (e.g., pupil, EDA, and fNIRS) that are not related to the same aspect of pain can enhance the accuracy of the system and provide better insights into the physical problem, this might not be possible to achieve with a single sensor modality^81^. Sensor *robustness* is another benefit of multimodal systems, since the use of a single sensor modality might react to a particular confounding factor, the overall reliability of the system might be affected. However, with the use of multiple sensor modalities, validation of sensor data can be achieved. For instance, if a patient normally suffers from high blood pressure (HBP), the use of a pulse-based sensor (e.g., PPG) might have a negative effect on the collected data, on the other hand, if other sensor modalities (e.g., EEG and EDA) are used in conjunction with PPG, the collected data will be more reliable as the other two sensing technologies can be used to validate the effect of HBP in the affected sensor. Finally, *multimodality* is not a new concept and has been naturally performed by animals and humans to assess different situations in the environment. For example, animals use a combination of multiple senses (e.g., vision, smell, hearing) to avoid risks or threats and improve their chances of survival^109^. Therefore, with the use of multiple modalities (i.e., different dimensions) to monitor the different physiological changes in pain, medical practitioners can obtain a better understanding of the pain experienced by the patients.

Obtaining quality sensor data plays a vital role in providing correct decision-making outcomes. In general, the better the quality of the sensors and more reliable the data, the more valuable it is. In applications of health monitoring, good quality data is imperative, as data not only helps patients receive better care, but also it makes for better research and analysis^110^. Three main categories of sensor quality can be observed in the literature, consumer-grade sensors, research-grade sensors, and medical-grade sensors. Although, *consumer-grade sensors* have gained popularity in healthcare applications due to their lower cost, data quality can be insufficient in healthcare applications. For instance, some fitness trackers (e.g., HRM-Tri by Garmin, FitBit PurePulse, Microsoft Band) explicitly acknowledge that their devices are not for medical use and should not be relied upon for detecting health conditions, including pain^111^. Another limitation of consumer-grade sensors is the lack of raw data availability and the inclusion of proprietary steps in their data analyses^112^. *Research-grade* sensors represent an option to obtain relatively good-quality data and with the flexibility to access the raw data, which allows the development and testing of own custom algorithms. This also allows for algorithms to be easily implemented across other similar devices, as well as transparency to estimate the outcome variable. On the other hand, *medical-grade sensors* can produce high-quality sensor data, higher measurement accuracy, higher sensitivity, and are more stable and robust^110^. A limitation of such high-quality sensors is their relatively high cost, as having to deploy many highly accurate but expensive sensors will occur in higher deployment costs. However, the use of medical-grade sensors results in less time spent in their maintenance and calibration, which might lead to reduced overall operational costs in the long run. Finally, it is important to consider the quality of sensing systems in healthcare, as decision-making outcomes, better diagnosis, and improved patient care depend on having accurate and reliable information.

### Analytical techniques for decoding pain

Most of the reviewed studies used classical machine learning (ML) models, however, they rely heavily on feature engineering. Although in many cases, ML models such as SVM, RF, and LR exhibited the best performance in twenty nine of the reviewed studies, the success of these models mostly depends on the feature engineering process. A clear challenge in the feature engineering process is the need of *domain knowledge* to create features that are relevant. For example, having knowledge of the typical physiological response exhibited by patients in pain can help in the identification of metrics that are more relevant and valuable for the problem at hand. However, this approach can be highly subjective and bias to the person’s creativity or expertise, which might result in missing potentially useful features that might be ignored^113^. During the feature engineering process, large number of features are often generated (e.g., applying mathematical/statistical functions to the same sensor data), which can make the ML suffer from the *curse of dimensionality*. This can lead to obtaining highly noisy features, correlated features (i.e., collinearity), and without significant benefit. This results in trying to solve more complex problems, decreasing ML model’s performance, and increasing computation cost^114^. In the reviewed literature, dimensionality reduction or feature selection techniques were implemented as a *feature optimisation* stage to remove irrelevant or correlated features, however, this process is often computational expensive. The feature engineering process is time consuming and it involves multiple steps, including the design of the features, test their efficiency with the model, modify some of features or try other features, and repeat the process until the model exhibits an acceptable performance. Overall, the feature engineering process should not be considered lightly as it plays a major role in determining the outcome of a ML model.

An alternative to the feature engineering problem is deep learning (DL). A common application of DL models is to automatically create candidate features directly from data. Automated feature engineering extends the concept of domain knowledge as DL leverages the use of multiple hidden layers to explore different connections and extract the best features to solve the learning problem. The multi-layer architectures used in DL are inspired in the process that take place in core sensorial regions within the human brain, in which the multi-layer data representation extracts low-level features in the first layers and high-level features in the last layers^115^. The main advantage of DL compared to ML models is that it automatically finds significant features without the need of feature engineering or human domain knowledge expertise. This approach has shown to be successful in different complex tasks and DL models have outperformed well-known ML techniques in several domains, including natural language processing, computer vision, bioinformatics, speech and audio processing, among others^116^. However, a key challenge in the success of DL models is the need for large amounts of training data, as the data increases, a well-behaved performance model can be obtained^115^. This in part may explain why most ML models outperformed DL models using the same dataset^35,39^ or with the use of the same sensor modality^117,118^. In many cases, large datasets (in particular, labelled datasets) may be too difficult or costly to be collected for many learning problems. A possible solution to overcome the need for large datasets is the use of retraining DL models, i.e., *transfer learning*^119^. Transfer learning aims at transferring the knowledge across different but related domains, this approach has shown that DL models already trained on a specific dataset and build to solve a specific task can be reused as the initial phase for training on a different dataset for a different task^120^. Although DL have achieved accuracies that are far beyond that of classical ML models in other domains, the need for large amounts of training data should be considered in DL applications for pain assessment.

Although there are some publicly available datasets for the assessment of pain, there is a need of datasets that combine neurological and physiological signals. Available datasets include videos of face^121–123^, physiological signals (EDA, sEMG, ECG) and videos of face^124^, and physiological signals (EDA, sEMG, ECG), audio signals, video of the body and face, and thermal video of the face^125^ in adults. There are also datasets from infants, including vital signs (Sp02,HR, RESP, BP) with videos of face^126^, and with cry signals alone^127^. However, these datasets do not explore neurological and physiological sensing technologies together. In the reviewed literature, the dataset containing EEG and EDA data^128^ was identified through the work of Sun et al.^129^, but only the EEG data was analysed. The lack of such datasets can also be reflected in the limited number of studies that were identified in this review that explored the combination of neurological and physiological signals. The combination of neurological and physiological information has been widely explored in other areas of research including cognitive workload^105,130^, neuroergonomics^131,132^, or learning and training^26,133^. In addition, recent studies in multimodal data fusion have found that the combination of multiple neurological sensing technologies such as EEG and fNIRS exhibited significantly better results in cognitive workload assessment tasks^134^. The combination of EEG and fNIRS offers a possible stream in pain research that has not been explored before. Therefore, the inclusion of neurological data in conjunction with physiological and behavioural data has the potential to improve the overall accuracy of the assessment systems, which also helps obtain a more complete understanding of pain that would be otherwise unavailable from neurological, physiological, or behavioural data alone.

There is a need for the adoption of context-aware systems that can use additional information (contextual information) to improve the performance of objective tools for pain assessment. In the paradigm of computing, contextual information can be defined as a set of real world parameters or information that can be used to characterise the situation of an agent (e.g., person, place, or physical or computational object)^135^. In this case, context could be, for example, the use of health records from the patient, socioeconomic information, patient’s disease, genetic or familial variables, or situational or emotional factors that can help (in addition to the neural and physiological information) to understand in a better way the patients’ pain sensation. In the reviewed literature, there are some studies^43,44,53^ in which physiological data, health records, and medication information were used in their assessment. While in the work of Fang et al.^136^, congenering information was employed to cluster patients based on their type of disease (e.g., pulmonary, renal, cardiovascular) and trained a dedicated model for each category, their method exhibited better results than training a single model with all patients combined. In another study, Kachele et al.^37^ designed a personalised model to focus on each individual rather than the whole group, which also showed improved classification accuracy. Overall, the use of contextual information can help improve the perception of patients’ pain and can serve as the basis of well-informed decisions, not only by the medical practitioners, but also, by autonomous systems to trigger actions upon particular circumstances.

With the use of multiple sensor data and rich contextual information, intelligent fusion architectures will be needed. The fusion architecture should be able to fuse different data from sensors as well as the additional contextual information. An advantage of the fusion architecture is *conflict resolution*, considering that the use of multiple input variable often leads to contradictions and inconsistencies in the data^109^. For example, if increased levels of HR and BP data are observed, which may indicate an episode of pain, but at the same time, no changes (e.g., increase/decrease) are detected in other parameters (e.g., PUPIL, EDA, or EEG) in the data, this event may create a conflict (e.g., a false positive) in the pain monitoring system. However, contextual information (e.g., health records) could indicate that the patient suffers from hypertension, therefore the intelligent fusion architecture will control this conflict and resolve it by using the contextual data. Another advantage of the intelligent fusion architecture is *weighing information*, since not all sensor data convey the same level of reliability, and in the event of pain, some sensors may provide information that has more value than others in certain circumstances^108^. For instance, if the patient is asleep and in pain, some metrics (e.g., pupillometry or movement) will not provide reliable information, but other measures (e.g., EDA, PPG, or RESP) will be more important and relevant to provide evidence of pain. Therefore, the fusion architecture will be able to apply individual weights with different divergence measures based on their relative importance depending on the time of day^137^. Eventually, the intelligent fusion architecture will be particularly useful in clinical settings where real-time decision making is imperative to deliver decision support to clinicians in the shortest amount of time.

### Practical implications on the use of sensors

Confounding factors represent a major challenge in pain monitoring using sensors. In the reviewed literature, the most common reported confounding factor within the physiological response was stress^31,33,34,40,43,49^. Pain and stress share conceptual and physiological similarities, and in both events, changes in all systems are expected, including cardiovascular, respiratory, nervous, and muscular systems^138^. In the cardiovascular system, for instance, acute stress leads to increased blood pressure, heart rate, and cardiac output. In the respiratory system, acute stress causes increased respiration rate and, thus, increased oxygen consumption. With regards to the musculoskeletal system, stress causes a reduction of skeletal muscle blood flood, which leads to contraction of muscles^139^. With regards to the effect on the nervous system, stress increases the activity of the sympathetic nervous system and decreases the activity of the parasympathetic nervous system^140^. Given the similarity of physiological responses in stress and pain situations, it is not surprising that these two are difficult to isolate from each other^13^; in fact, this might indicate one of the reasons for misclassification by the learning models. In addition, pain can be considered a physiological stressor in the field of stress research^141^. Similar to pain, stress is a human response to physical or emotional strain and is a reaction that threatens homoeostasis (i.e., maintaining a balanced internal environment)^142^. The persistence of any stressor often leads to compromised well-being and chronic long-term suffering (e.g., chronic pain)^143^. Individuals can also report acute stress as pain. Other confounding factors identified in the literature, that are directly related to pain, are fear and anxiety. Finally, by recognising the role of confounding variables in pain processing, the scope of pain could be expanded with additional valuable information that will help us broaden our understanding of pain^142^.

A central challenge in establishing an objective and reliable assessment of pain, using sensing technologies, is variability—both intra- and inter-individual variability—in the experience of pain. By definition, pain is not only a subjective experience, but also a highly personal one^144^. First, intra-individual differences exist within the same person and are often observed across repeated observations at different times (e.g., morning vs evening) or in different situations (e.g., after anaesthetics, or during different emotional or cognitive states). Intra-individual differences affect pain responses within the same person, which reflects that pain experience is highly influenced by a combination of different factors unique to the person, making the pain experience completely individualised^103^. In this context, pain is fundamentally dynamic rather than static, with individuals reporting pain intensity levels varying considerably during different time periods ranging from moments to hours to days^145^. Intra-subject variability is considered to be lower than inter-subject variability^146,147^. Second, inter-individual variability is generally observed among individuals due to differences in gender, age, ethnicity, and psycho-social processes, among others^144,148^. In the case of pain experience, inter-individual differences have been exhibited by broad pain variability between individuals as a response to experimentally induced pain^149^. In automated pain assessment, these differences have shown an adverse effect in the modelling of both neural and physiological signals for the assessment of pain. In the reviewed literature, several studies using neural (EEG, fNIRS) or physiological (PPG, EDA, EMG) signals reported notable inter-subject variability in pain perception and responses; these reported differences in neural and physiological responses affected the capacity of machine learning models to generalise across people^28,34,70,71,103,117^. Inter-individual differences often lead to model overfitting, which can be interpreted as the lack of model’s generalisability to give accurate predictions with new data^117^. In addition, intra-individual variability is usually superimposed on top of inter-individual variability, which represents an added confounder to model neural and physiological signals^150^. Both intra- and inter-individual variability in pain perception should be considered in the analysis of neurophysiological signals when developing pain recognition models.

Therefore, there is a need to build robust analytical methods to decode pain in the presence of inter- and intra-subject variability. For instance, in several studies, inter-individual variability in neural or physiological signals is minimised by a *standardisation* strategy (e.g., linear scaling, log scaling, *z*-score, etc.)^34,37,103,107^. This strategy helps to improve the accuracy of machine learning models trained on a group of individuals and applied on a different individual (i.e., subject-independent models), which is desired in most practical applications^103^. Another option is capturing an *extensive dataset for each individual* and, then, retraining a learning model based on that individual dataset (i.e., subject-dependent models) to incorporate individual factors, which can serve as a tailored approach. However, collecting large amount of data per individual is often not sufficient to design a robust model and this method is less practical in real clinical settings. While, some studies have addressed the inter-individual variability in pain assessment, little has been done to simultaneously address the challenges of intra- and inter-individual variability. For instance, Lopez et al^70^. investigated the use of *multi-task learning*, a type of transfer learning in which a personalised learning model that account for individual differences in physiological responses to pain, while the model is trained on the entire dataset. This method uses a soft-clustering mechanism that enables the model to determine the similarities between the individuals in the dataset and to identify the number of clusters representing groups of individuals (i.e., tasks) with common similarities. In Pourshoghi et al.^71^ and Pouromran et al.^28^, another *clustering* approach based on k-means was employed, in which population data was clustered and cluster-specific models were built. In this method, data collected from an individual is allocated to the closest cluster to then use the cluster-specific model to assess pain intensity. It is worth mentioning that in the study done by Pouromran et al.^28^, EDA signals exhibited significantly better results in the cluster-specific models compared to EMG and ECG signals. These results implied that the EDA signals appeared to be more comparable among different individuals, while EMG and ECG signals presented substantial inter-subject variability in response to pain. While automated and objective pain assessment is still a challenge, designing learning models based on similar groups of neural and/or physiological response to pain could be useful to reduce intra- and inter-individual variability.

There are also opportunities to use an objective assessment of acute pain to assist in the diagnosis of chronic pain. In general, acute pain can be considered less complex to be assessed since it has a specific, treatable cause (e.g., broken bone, torn ligament) and is generally sudden and intense. For instance, acute pain typically originates from a response to an injury, illness, trauma, or medical procedure. Acute pain lasts for a short period of time, and often ceases when the underlying problem has healed. When pain persists longer than expected, beyond the expected time for healing, it is commonly referred to as chronic pain^151^. Chronic pain can continue even after the injury or illness that caused it has healed or gone away, with pain signals remaining active for months or years. Chronic pain includes conditions such as complex regional pain syndrome, phantom limb pain, chronic low back pain, and fibromyalgia syndrome. It is unlike acute pain, which plays a protective role by eliciting motivation to minimise harm. Rather, chronic pain is considered a disease in itself^152^. Some people experience chronic pain even with no previous illness or apparent trauma. The assessment of chronic pain is complex and often comprises several domains, including physiological indicators and contributing factors, with physicians and other clinicians assessing patients for function, quality of life, mental and emotional health, and factors that aggravate or alleviate pain. In addition, the chronic pain data captured by neurophysiological devices will not be as responsive and conclusive as that of acute pain^29^. Chronic pain is, therefore, considered a complex problem, multifaceted, with multifactorial causes^153^. In this context, the assessment of acute pain can facilitate early diagnosis, monitoring of disease progression, and overall effective therapeutic evaluation^154^. In addition, pain assessment serves other important functions in the management of pain such as, documenting the intensity and severity of the pain condition, tracking the course of pain in time, and providing mechanistic information^155^. Therefore, reliable and valid assessments of pain can generate important historical information that can assist clinicians in identifying patients who may develop persistent pain and, thus, in designing early interventions for the management of chronic pain.

There are opportunities to use neurophysiological sensors to estimate pain in diverse applications. A system that serves not only to diagnose pain, but also has the ability to *identify the location of pain* on the body by measuring changes in neurophysiological response can be of benefit to patients unable to self-report. In the reviewed literature, only two studies explored the possibility to find the location of pain, using EEG^64^ and fNIRS^96^, with diverse results. Some studies using physiological data alone suggested that it is not possible to find the location of pain without the use of neurophysiological sensors (e.g., EEG, fNIRS)^29,32^. In this context, Hu et al.^96^ used augmented reality (AR) as a visualisation interface, which can help clinicians determine when and where the patients are suffering from pain. AR offers the advantage to be used as smart glasses, in contrast to tablets or smartphones, which allows clinicians to be hands-free to perform other tasks. Another possible application is the use of a pain assessment system as a means of *biofeedback* in physical rehabilitation tasks. For instance, in guiding the intensity of physical rehabilitation to identify the efficacy of treatment and to decrease the risk of re-injury, as well as in helping to design programs tailored to the specific pain sensitivity of each patient^156^. In this regard, Badura et al.^98^ designed a study to monitor pain in patients during fascial therapy, with the intention to use it as real-time feedback on the intensity of the therapy, to avoid any tissue damage, and to improve therapy outcomes. Another opportunity to the use of neurophysiological indicators of pain is guiding audiologists in finding the most *suitable stimulation level* for each patient in cochlear implants. The neurophysiological indicators have the potential to provide an objective measure to inform audiologist whether the electrical stimulation of the cochlea is comfortable to the patient and, therefore, not too loud or uncomfortable. In addition, it will help to guide post-implant programming as cochlear implants need to be reprogrammed frequently to ensure they convey the sound information to the auditory nerve^157^.

This literature review presents some limitations. The choice of databases for article search may be a possible limitation of this reviewed. Although, we used three well-known databases, it could be argued that studies have been missed. However, to mitigate this, we searched for other articles in the reference list of the identified studies. In addition, there are studies that used facial expressions based of images or videos in combination with physiological sensors, in particular those studies using available datasets, such as the Biovid and X-ITE datasets. Many of these studies were not included because, in many instances, a separated analysis of the physiological data alone was not presented. Those identified studies that presented a separated analysis on the physiological data were included.

To conclude, pain is a complex and subjective experience that presents diverse measurement challenges. Despite the difficulty inherent in measuring an individual’s experience of pain, there are different sensing technologies that can be used as a surrogate measure of pain. Currently, there is no valid and reliable metric of objectively quantifying an individual’s pain experience. Therefore, the field of pain management would benefit greatly from an objective, neurophysiological marker of pain^14^. In this work, we aim to conduct a systematic review of the published literature to identify relevant non-invasive sensing technologies that can be used for the assessment of human pain in real-time applications. In this context, three main research questions (please refer to “Methodology” section for more details) were defined and the main findings are presented in the following paragraphs.

**Q1. What sensors can be used to quantify an individual’s pain experience?** Two main types of non-invasive sensors were identified in the reviewed literature: neurological (fNIRS, EEG) and physiological (EDA, sEMG, ECG, PPG, Resp, Pupil, and SKT). Among these sensing technologies, EDA, EEG and ECG were the most popular in the literature (refer to Fig. 2). While the majority (two-thirds) of studies used a single sensor, the use of different sensor modalities (i.e., multimodality) provides more measures for different dimensions of pain. Multiple sensing modalities presents a more complete understanding of pain, that would be otherwise unavailable from a single sensor. In addition, the use of a single sensor modality presents different disadvantages against multimodal sensing including, low reliability due to sensor failures, uncertainty to data quality, and low sensitivity to capture a complete understanding of the individual’s experience of pain. Quality sensor data not only offers more value for research and analysis, but also allows better decision-making and diagnosis.

**Q2. What analytical techniques are used for the decoding of pain?** Most of the reviewed studies used classical machine learning (ML) models. From those studies, support vector machines (SVM), random forest (RF), and logistic regression (LR) exhibited the best individual performance in twenty six of the reviewed studies. However, classical ML models rely on the generation of hand-crafted features, which can limit the performance of the models. On the other hand, deep learning (DL) models can automatically obtain features directly from data. Nevertheless, DL models require large amounts of training data. Although, there are several available datasets, there is no dataset that combines both neurological and physiological sensors. In addition, contextual information such as health records, genetic and familial data, situational or emotional factors have the potential to improve learning models in decoding an individual’s pain experience.

**Q3. What are the practical implications on the use of sensors in the assessment of pain?** Confounding factors represent a major challenge in the application of sensors for the assessment of pain. Stress is the most common confounding factor in the reviewed literature, stress shares conceptual and physiological similarities with pain that make these two difficult to isolate from each other. In addition, intra- and inter-individual variability should be considered when designing learning models to decode pain from sensing technologies, since these often affect the capacity of learning models to generalise across people. Finally, there are also opportunities to use an objective assessment of acute pain to assist in the treatment of chronic pain and help clinicians to identify earlier individuals who may develop persistent pain.

## Methodology

### Research questions

The aim of this literature review is the identification of relevant sensor technology that can be used for the objective assessment of human pain. In this context, two main technologies are of interest, the type of sensors that can be used for the assessment of pain, and the data modelling techniques (i.e., machine learning, deep learning) that are implemented for the recognition of pain. It is also of interest, to understand the main implications in the application of these technologies for the design of a real-time monitor that could assist medical practitioners in the assessment of pain in non-verbal populations. A summary of the main research questions of this review are as follows^158^:

- Q1. What sensors are used for the assessment of pain?
- Q2. What analytical techniques are used for the decoding of pain?
- Q3. What are the practical implications on the use of sensors in the assessment of pain?

### Search strategy

This review was performed according to the Preferred Reporting Items for Systematic Reviews and Meta-Analyses (PRISMA) methodology^159^. The PRISMA method is based on four stages that fall under the scope of the review: Identification, screening, eligibility checking, and selection. A keyword search was completed in PubMed, Web of Science, and Scopus in July 2022. Search terms were used in a combination, using variations of the keywords including in the following two groups: ((machine learning OR deep learning OR artificial intelligence OR automatic) AND (pain AND (assess* OR measure* OR intensity OR scale OR recognition))). In addition, the reference lists in the identified studies were examined to find additional publications of interest (i.e., snowballing).

### Inclusion and exclusion criteria

Studies that met all of the following criteria were included in the review: (1) peer-review publication in the English language; (2) studies published within the last decade (January 2013–July 2022); (3) studies that conducted objective pain assessment by using at least one sensor to measure neurological (e.g., EEG, fNIRS), physiological sensors (e.g., HR, EDA, PPG), and/or their combination; (4) the objective of the study should be related to pain assessment or pain recognition applying machine learning, deep learning, or artificial intelligence; (5) methods that report the effectiveness of the models (e.g., accuracy, mean absolute error) in identifying pain (e.g., numerically or categorically).

Studies were excluded from the review if they met any of the following criteria: (1) use of sensor technologies that are: not portable, not cost effective, or impractical for the design of a bedside monitor; (2) technologies that are still in a proof of concept stage; (3) studies that present invasive methods for pain assessment; (4) studies that present protocols for pain assessment; (5) studies that based their analysis on the use of video recognition, facial expressions, gesture, posture, behaviour, or voice analysis; (6) studies that focus on fibromyalgia, chronic, neurogenic, or neuropathic pain; and (7) letters to the editor, commentaries, or abstract-only publications.

## Data Availability

The data that support the findings of this study are available from the corresponding author upon reasonable request.
